# An insight on the impact of teleost whole genome duplication on the regulation of the molecular networks controlling skeletal muscle growth

**DOI:** 10.1371/journal.pone.0255006

**Published:** 2021-07-22

**Authors:** Bruno Oliveira Silva Duran, Daniel Garcia de la serrana, Bruna Tereza Thomazini Zanella, Erika Stefani Perez, Edson Assunção Mareco, Vander Bruno Santos, Robson Francisco Carvalho, Maeli Dal-Pai-Silva

**Affiliations:** 1 Department of Histology, Embryology and Cell Biology, Institute of Biological Sciences, Federal University of Goiás (UFG), Goiânia, Goiás, Brazil; 2 Department of Structural and Functional Biology, Institute of Biosciences, São Paulo State University (UNESP), Botucatu, São Paulo, Brazil; 3 Department of Cell Biology, Physiology and Immunology, Faculty of Biology, University of Barcelona, Barcelona, Spain; 4 University of Western São Paulo (UNOESTE), Presidente Prudente, São Paulo, Brazil; 5 Fisheries Institute (IP-APTA), São Paulo, São Paulo, Brazil; University of Minnesota Medical School, UNITED STATES

## Abstract

Fish muscle growth is a complex process regulated by multiple pathways, resulting on the net accumulation of proteins and the activation of myogenic progenitor cells. Around 350–320 million years ago, teleost fish went through a specific whole genome duplication (WGD) that expanded the existent gene repertoire. Duplicated genes can be retained by different molecular mechanisms such as subfunctionalization, neofunctionalization or redundancy, each one with different functional implications. While the great majority of ohnolog genes have been identified in the teleost genomes, the effect of gene duplication in the fish physiology is still not well characterized. In the present study we studied the effect of WGD on the transcription of the duplicated components controlling muscle growth. We compared the expression of lineage-specific ohnologs related to myogenesis and protein balance in the fast-skeletal muscle of pacus (*Piaractus mesopotamicus*—Ostariophysi) and Nile tilapias (*Oreochromis niloticus*—Acanthopterygii) fasted for 4 days and refed for 3 days. We studied the expression of 20 ohnologs and found that in the great majority of cases, duplicated genes had similar expression profiles in response to fasting and refeeding, indicating that their functions during growth have been conserved during the period after the WGD. Our results suggest that redundancy might play a more important role in the retention of ohnologs of regulatory pathways than initially thought. Also, comparison to non-duplicated orthologs showed that it might not be uncommon for the duplicated genes to gain or loss new regulatory elements simultaneously. Overall, several of duplicated ohnologs have similar transcription profiles in response to pro-growth signals suggesting that evolution tends to conserve ohnolog regulation during muscle development and that in the majority of ohnologs related to muscle growth their functions might be very similar.

## Introduction

Fish skeletal muscle represents up to 60% of total body mass in some species, being the most abundant tissue, responsible for their underwater propulsion and with a key role on homeostasis by acting as a protein reservoir among other functions [1, 2].

Muscle growth is a multifactorial process that incorporates intrinsic and extrinsic signals in its regulation, with fibre size as a result of the balance between protein synthesis and degradation being shifted towards net protein accumulation and the activation and differentiation of myogenic progenitor cells (MPCs). In juvenile and adult fish, the MPCs are located in the basal lamina of the muscle fibres and are known as satellite cells (SCs) [3]. Under normal circumstances the SCs are in a quiescent stage but become activated in response to injury or pro-growth signals (such as growth factors) or nutrients (such as amino acids). Once activated, the SCs become proliferative myoblasts that eventually can either fuse to each other to form new fibres (hyperplasia) or to pre-existent fibres, increasing their size to maintain the nuclei/cytoplasm ratio (hypertrophy) [4–6]. Multiple molecular networks regulate SCs activation and differentiation [4, 5] with the myogenic regulatory factors (MRFs), a family of basic helix-loop-helix (bHLH) transcription factors that includes MYOD, MYF5, MYOG and MYF6, as one of the main regulators of the process. These factors are sequentially expressed and direct the myoblasts towards differentiation and muscle fibre formation [7, 8], a process also regulated by MEF2. MEF2 represents a family of transcription factors that acts synergistically with MRFs to activate muscle-specific transcription, mostly enhancing the expression of differentiation genes [8–11]. In fact, Hinits and Hughes (2007) showed that knockdown of MEF2 proteins in zebrafish (*Danio rerio*) reduced the mRNA levels of sarcomeric proteins, prevented sarcomere assembly and blocked muscle function [12].

Growth factors and amino acids have demonstrated to play an essential role in promoting protein synthesis, indicating the coordination of both endocrine and nutritional inputs to regulate muscle growth [13–15]. The phosphorylation of TORC1 (the complex formed by mTOR and RAPTOR) promotes phosphorylation of S6K and 4EBP1, stimulating protein synthesis at the same time that inhibits degradation, increasing protein accumulation [16, 17]. Phosphorylation of TORC1 can happen through two different pathways, the IGF-PI3K-AKT axis stimulated by the insulin-like growth factors (IGF1 and IGF2) and the LAMTOR-RAG complex activated by amino acids [18–20]. IGF1 and IGF2 are major regulators of skeletal muscle growth, in a system that also includes several receptors (IGFRs) and different binding proteins (IGFBPs). Many studies have investigated the effects of IGFs treatment in fish muscle cells, with important roles on protein synthesis [13, 14, 21], myogenesis [22–24] and metabolism [25, 26]. Muscle growth is strongly dependent of mTOR signalling, activated by IGFs through increase of AKT phosphorylation [13, 27]. TORC1 blocking impaired fish muscle cellularity, growth performance and down-regulated the expression of *igf1*, *igf2* and *igfbps* [28]. Studies also showed increased mTOR phosphorylation due to amino acids treatments or availability [13, 16, 29]. Seiliez et al (2008) observed enhanced phosphorylation of AKT, mTOR and the downstream targets S6K and 4EBP1 after meal feeding to rainbow trout (*Oncorhynchus mykiss*) [16], while amino acids alone stimulate mTOR phosphorylation and the expression of many genes in the IGF pathway in Atlantic salmon (*Salmo salar*) and gilthead sea bream (*Sparus aurata*) muscle cells [13–15, 29, 30]. The mechanism by which amino acids stimulate protein synthesis by themselves involves the LAMTOR-RAG complex, which is required for the amino acid-induced re-localization and activation of TORC1 in the lysosomes [18, 31, 32]. In fact, piglets infused with amino acids showed increased abundance of several LAMTOR-RAG components in the skeletal muscle [33].

In addition to protein synthesis, IGF-PI3K-AKT pathway reduces protein degradation in teleost, through phosphorylation of FOXO transcription factors and consequent inhibition of the atrogenes TRIM63/MURF1 and FBXO32/MAFBX [34, 35]. Protein degradation and turnover are significantly regulated by Myostatin (MSTN), an important member of TGFB family, expressed and secreted predominantly by skeletal muscle and widely known as a negative regulator of muscle growth [17]. The Follistatin (FST) is a general inhibitor of TGFB molecules, especially myostatin, whose gene disruption correlates with increased muscle mass in rodent [36–38], cattle [39, 40] and fish [41, 42]. MSTN triggers muscle protein degradation through binding to the activin receptor and consequent phosphorylation of SMAD transcription factors, while FST promotes nearly-irreversible binding and neutralization of activin [17, 43, 44]. In addition to influence on protein accumulation, MSTN also showed roles in fish myogenesis, inhibiting the proliferation of rainbow trout myoblasts, but not the differentiation [45].

In teleost fish, the molecular networks have gone through an expansion of some of its components due to an extra teleost whole genome duplication (TWGD) event in the base of the lineage 350–320 million years ago [46]. The TWGD was followed by a process of re-diploidization where the majority of WGD-duplicated genes, also called ohnologs [47–49], were lost through pseudogenization, with 15–21% of them retained through different molecular processes such as neofunctionalization, subfunctionalization or redundancy [50]. During subfunctionalization each duplicate gene acquires part of the regulatory elements of the ancestral pre-duplicated gene, which usually culminates in a shared regulation of a biological process. During neofunctionalization one of the ohnologs acquire a different regulatory elements that will eventually lead the acquisition of a new function, different from the ancestral gene [51]. Extra copies increases gene product that might provide some evolutionary advantage, leading to the retention of duplicated genes with common functions and regulatory elements [52]. Previous studies have estimated the percentage of teleost ohnologs differentially retained between the teleost superorders Ostariophysi and Acanthopterygii, known as lineage-specific paralogues [50]. However, due to their specific WGD origin, we will refer them as lineage-specific ohnologs (LSOs) from now on. Several of these LSOs are components of the myogenesis and protein synthesis/degradation networks [50].

Ohnolog genes represent a challenge to understand the molecular regulation of fish muscle growth and development. Despite some authors have suggested that redundancy is an important mechanism in the retention of duplicated components of molecular pathways related to development and signalling [52–54], this possibility has not been fully explored. Normally, conservation or divergent functions of ohnolog genes are studied by comparison against non-duplicated orthologues, generally from studies in mammalian models. While it is true that functions can be very well conserved through evolution, comparisons between lineages that are so evolutionary distant could lead to misleading conclusions.

In the present study we wanted to gain insights on the role that teleost WGD might have on the transcription of ohnolog genes involved on the molecular networks controlling muscle growth, and try to estimate the impact of neofunctionalization, subfunctionalization and redundancy on their regulation. To achieve these objectives and to correctly interpret the results obtained we used LSOs differently retained between Ostariophysi and Acanthopterygii superorders. The working premise is that the singleton (not duplicated gene) in one of the lineages would retain the functions of the ancestral gene (pre-WGD) while LSOs would have gone through some retention mechanisms that would leave a signature on their transcription profiles in response to different stimuli. To further improve comparison, we used two species of teleost occupying similar ecological niches (warm climate, fresh water and comparable diets) but from the two different superorders: the pacu (*Piaractus mesopotamicus*; Ostariophysi) and the Nile tilapia (*Oreochromis niloticus*; Acanthopterygii) [55, 56]. Juveniles from both species were used on a fasting-refeeding experiment to manipulate growth rate [57–59].

## Material and methods

### Lineage-specific ohnologs (LSOs) identification

For the identification and verification of LSOs, peptide sequences of 238 components of the myogenesis and IGF-AKT-mTOR networks were retrieved from Ostariophysi (*Danio rerio*, *Ictalurus punctatus* and *Astyanax mexicanus*) and Acanthopterygii (*Gasterosteus aculeatus*, *Oreochromis niloticus*, *Takifugu rubripes* and *Tetraodon nigroviridis*) species (S1 File) (https://www.ensembl.org). For the pacu sequences, Ostariophysi orthologues were used as query (tBlastn) against the existent pacu skeletal muscle transcriptome (ENA accession number PRJEB6656) [60]. In order to verify their TWGD origin, Bayesian-based phylogenetic analysis was conducted. To this end, peptides sequences were aligned using GUIDANCE2 [61] with MAFFT as multiple sequences alignment protocol and only aligned regions with GUIDANCE2 score over 0.95 were used for phylogenetic tree reconstruction. MEGAX [62] software was used to estimate the most appropriate evolutionary model for each individual alignment, through best-fit model selection tool. Bayesian-based phylogenetic trees were constructed using BEAST2 [63] with 10,000 random seeds. Final trees were constructed using TreeAnnotator [64] with a 10% burning and visualized using FigTree v.1.4.2 (http://tree.bio.ed.ac.uk/software/figtree/) (S2 File).

### Ethics statement and experimental design

All procedures were performed in accordance with the Ethical Principles in Animal Research adopted by the Brazilian College of Animal Experimentation (COBEA) and the U.K. Animals (Scientific Procedures) Act 1986 (Home Office Code of Practice. HMSO: London January 1997). The protocol was approved by the Ethics Committee on Animal Use (protocol number 705) of the Institute of Biosciences, São Paulo State University (UNESP, Botucatu, São Paulo, Brazil) and following the ARRIVE guidelines [65]. Juvenile pacus and tilapias were obtained from the São Paulo Agency for Agribusiness Technology (APTA) (Presidente Prudente, São Paulo, Brazil). Fish were kept in 0.5m^3^ storage tanks and common garden conditions at 28°C, natural photoperiod (12 light: 12 dark) and fed twice a day with the same diet (Acqua Line Supra, Brazil). After the acclimation period, animals were fasted for 4 days followed by 3 days refeeding. Before sampling, animals were euthanized with an excess of benzocaine (≥250mg/L; Sigma-Aldrich, USA), fast-skeletal muscle was extracted and stored at -80°C until further analysis. Samples were obtained daily during fasting period (-4d, -3d, -2d and -1d), before refeeding (0d) and at different time points after refeeding started (6h, 12h, 1d, 2d and 3d). As biological replicates, we used 6 pacus and 6 tilapias in each experimental group.

### RNA extraction, reverse transcription and primer design

Total RNA was extracted from skeletal muscle samples using TRIzol® (Thermo Fisher Scientific, USA), following the manufacturer’s recommendations. Total RNA was quantified by spectrophotometry using the NanoVue™ Plus (GE Healthcare, USA) and its integrity evaluated by capillary electrophoresis in a 2100 Bioanalyzer (Agilent, USA), with all samples having 260/280nm and 260/230nm ratios above 1.8 and RNA integrity number (RIN) >7.0. A total of 1μg of RNA was reverse transcribed using the High Capacity cDNA kit (Thermo Fisher Scientific, USA), including a genomic DNA wipe-out step.

Primers were designed using Primer3 [66] (S3 File). MAFFTv7 was used to align LSOs and singletons to identify regions with low similarity for primer design. All primers were designed to work at 60°C and amplify 50-200bp regions expanding exon-exon boundaries when possible. Potential hairpins, self-dimers or cross-dimers were estimated using NetPrimer software (Premier Biosoft, USA).

### Gene expression

All qPCR were compliant with the Minimum Information for Publication of Quantitative Real Time (MIQE) guidelines [67]. Each qPCR reaction contained 8μL of diluted cDNA (1:40), 4μL of GoTaq® qPCR Master Mix (Promega, USA) and 3μL of 500nM forward/reverse primer mix. The reactions were performed in duplicates (technical replicates), under the following conditions: 95°C 10 minutes, 40 cycles at 95°C 15 seconds and 65°C 1 minute, in a QuantStudio™ 12K Flex Real-Time PCR System (Thermo Fisher Scientific, USA). Primer specificity was confirmed by the presence of a single-peak dissociation curve. Relative expression was estimated using the 2^-ΔΔCt^ method [68]. Three genes *ribosomal protein l13* (*rpl13*), *ribosomal protein l19* (*rpl19*) and *peptidylprolyl isomerase aa* (*ppiaa*) were selected for normalization of expression after stability was tested using geNorm software [69].

Principal Components Analysis (PCA) plot of the gene expression were constructed using ClustVis (https://biit.cs.ut.ee/clustvis/) [70]. PCA were calculated using the Singular Value Decomposition (SVD) with imputation and row scaling was obtained by Unit Variance Scaling method, which divides the values by standard deviation. Prediction ellipses were generated around groups with confidence level at 0.95. Heatmaps correlating genes transcription profiles were created using the Morpheus software [71]. The hierarchical clustering was performed using One Minus Pearson Correlation as metric and average linkage method. Non-hierarchical K-means clustering, obtained using the same metric, was used to better define different clusters of genes among the expression data.

### Statistical analysis

Statistical analyses were performed using RStudio v1.0.136 [72]. Normality was estimated using Shapiro-Wilk; when normality was not achieved, data was transformed using the Box-Cox approach [73] and re-analysed. When normality assumption was achieved, data was further analysed by a one-way ANOVA followed by post hoc Tukey-HSD test. When normality assumption was not fulfilled, a Kruskal-Wallis followed by Dunn’s post hoc test was applied. Correlation index and statistical significance between gene expression profiles were estimated using a Pearson correlation test. Graphs were constructed using ggplot2 R package [74].

## Results and discussion

After screening all the duplicated components of the IGF-AKT-mTOR and myogenesis gene networks, we identified 20 LSOs (11 in Ostariophysi and 9 in Acanthopterygii), with 5 related to myogenesis and 15 to the IGF-AKT-mTOR network (S1 File). Phylogenetic analysis showed that duplicated genes were grouped in two distinctive clusters related to fish species (S2 File) following a typical topology associated to TWGD duplicated genes [50]. Because some of the Acanthopterygii LSOs and singletons were not annotated specifically on the ensembl database we used specific nomenclature based on their phylogenetic relationship (S2 File).

**Fig 1:**
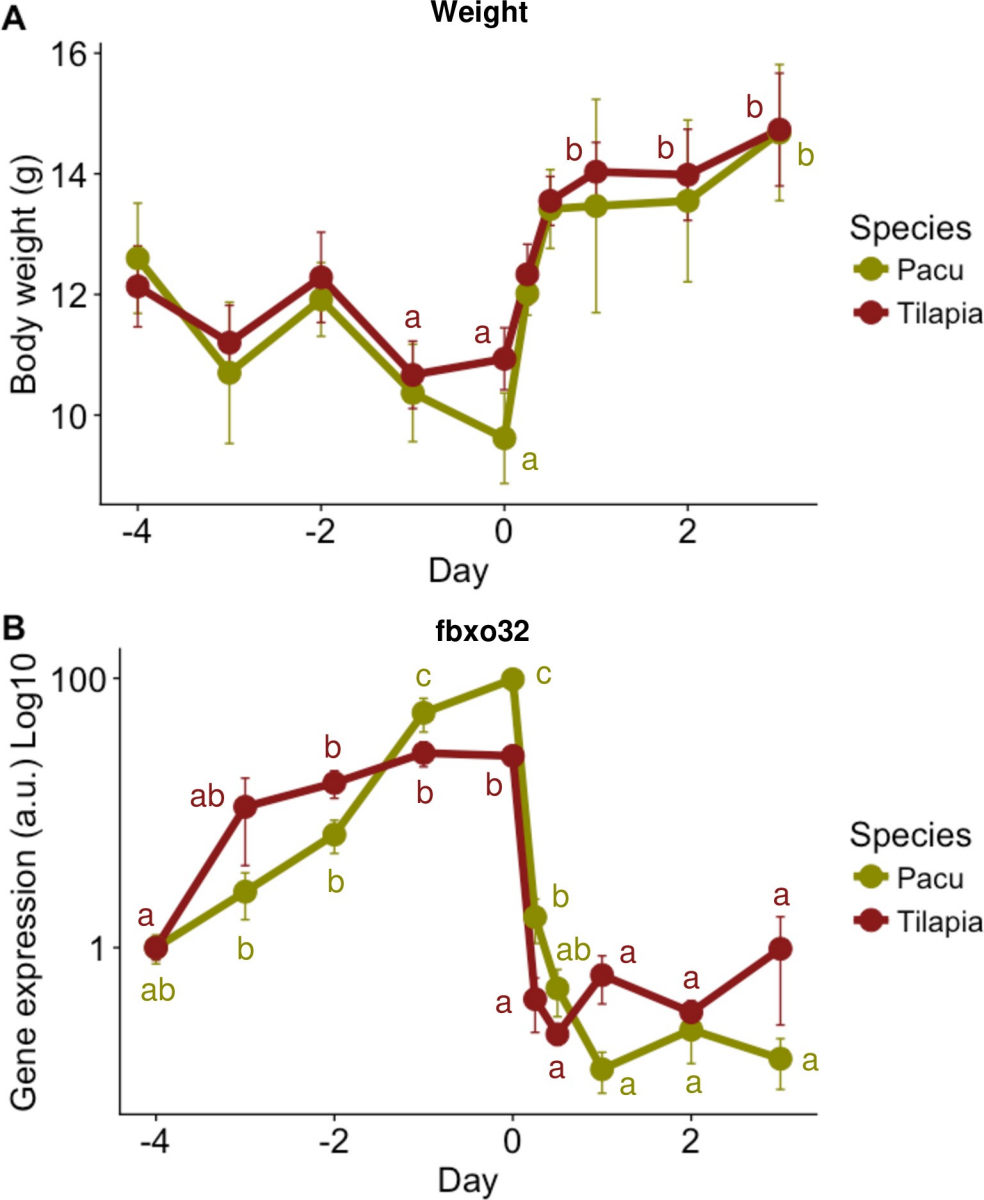
Response to fasting-refeeding protocol in pacus and Nile tilapias. **(A)** Evolution of total body weight (g) in pacu (green line) and tilapia (red line) juveniles during 4 days of fasting (-4 to 0 day) and 3 of refeeding (0 to 3 day). Values are shown as Mean±SE (n = 6), different letters indicate significant differences between time points for each species (P<0.05). **(B)** Relative gene expression of F-box protein 32 (*fbxo32*) in pacu and tilapia fast skeletal muscle in response to fasting-refeeding expressed in Log10 scale. Values are shown as Mean±SE (n = 6), different letters indicate significant differences between time points for each species (P<0.05).

Fish body weight decreased significantly between 10 to 22% during the period of fasting for both species, followed by a significant increase around 25 to 35% during refeeding (Fig 1A), indicating that growth was slower during fasting while increased during re-feeding [58, 59]. However, no significant differences on body weight were found between the individuals from both species in any of the timepoints recorded. Effectiveness of the protocol was further confirmed by determine *fbxo32* expression. The FBXO32 is a muscle specific E3-ubiquitin ligase involved on muscle protein degradation and atrophy [75] whose transcription is strongly modulated by nutrition in fish, being a well-known and much characterized marker of fasting-refeeding [15, 59, 76–78]. For both species, *fbxo32* transcription increased during fasting (26–98 fold-change range; P<0.001), concomitant with weight loss, to rapidly decrease after refeeding started (>150 fold-change; P<0.001) (Fig 1B) and growth and protein synthesis were recovered. Due to body weight and size were similar between species even during the fasting-refeeding protocol we can assume that differences at transcription between LSOs and singletons were not originated by differences in growing dynamics (due to both were equivalent).

**Fig 2:**
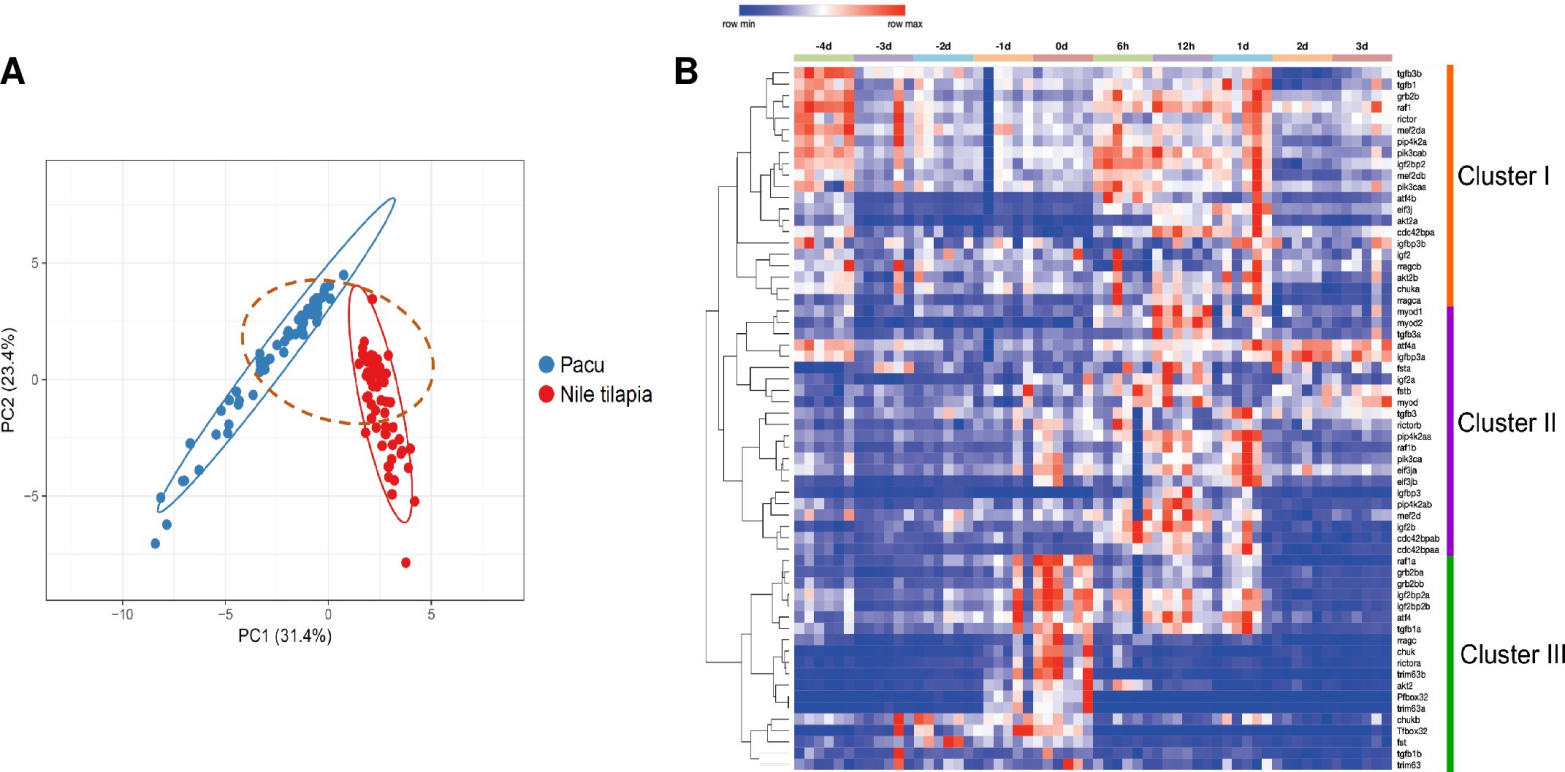
Lineage-specific ohnologs (LSOs) expression between skeletal muscle of pacus and Nile tilapias. **(A)** Principal component analysis (PCA) showing LSOs expression data grouping according to fish species. Principal components were calculated using the SVD with imputation and Unit Variance Scaling method for row scaling. Prediction ellipses have 0.95 of confidence level. **(B)** Hierarchical clustering and non-hierarchical K-means clustering (K-means = 3) for pacus and tilapias LSOs and singletons transcription in response to fasting-refeeding. Red indicates high and blue indicates low expression values. Heatmap shows the 2^-ΔCt^ values of LSOs expression and One Minus Pearson Correlation was used as metric for clustering.

The PCA analysis showed two distinctive clusters for Pacu and Tilapia LSOs (Fig 2A), suggesting some differences at transcription level between the species. However, the majority of genes on the PCA plot appeared relatively close to each other (dotted line on Fig 2A) and only the first component (that explains a 31.4% of the variability) was able to discriminate between the two groups, suggesting that the differences on expression for the majority of genes analysed between species might be relatively small. Closeness on the PCA analysis point out to gene redundancy or early stages of subfunctionalization, a possibility that was further investigated using hierarchical clustering and correlation analyses.

The hierarchical cluster analysis showed three distinctive groups of genes (Fig 2B). The first group (Cluster I) was formed by genes showing high transcription before the fasting period, to later be down-regulated during fasting and rapidly up-regulated during 24h after food intake was restored. A second group (Cluster II) was formed by genes whose transcription was stable during the fasting to rapidly increase 24h after refeeding, with some genes maintaining a relatively high transcription up to 3 days after refeeding. A third cluster (Cluster III) was formed by genes with a low level of expression that increased in response to fasting and rapidly decreased after food intake. Similarly to previous studies, in both species the components of the IGF-AKT-mTOR network and LAMTOR-RAG complex such as *igf2*, *rictor*, *rragc* or *raf1* had low transcription during the majority of the fasting period but rapidly increased when food intake was restored, concomitant to an increase on growth and protein synthesis [58, 59, 79, 80]. Genes involved in the control of myogenesis increased in response to food intake similar to previous studies [58, 59, 79, 80], indicating activation of SCs by genes such as *myod* and *mef2d* [11, 81, 82] and promotion of muscle growth by *fst* (via blocking myostatin, an inhibitor of muscle growth) [38, 42]. In addition, genes linked to protein degradation such as *trim63/murf1* [83, 84] were among those up-regulated in response to fasting as reported in previous studies [15, 85].

Overall, the response to fasting-refeeding was very similar between the two species, as we would expect for conserved functions on protein synthesis and muscle development. However, our data also gave very interesting insights how ohnolog genes related to growth might diverged after WGD. Hierarchical clustering topology (Fig 2B) and correlation analysis between LSOs and singletons (Table 1) gave us some indication of the molecular mechanisms behind the ohnologs retention and how they have affected their transcription. We took into consideration the correlations between the pair of LSOs (LSO-LSO) and between the LSOs and the single gene (LSOs-singleton), and also the position of LSOs and singleton among the heatmap clusters. We considered a possible redundancy when both LSO-LSO and LSOs-singleton correlations were significant (likely have common regulatory elements) (Table 1), and all these redundant genes showed the two LSOs clustering together in the heatmap (Fig 2B). We considered a possible subfunctionalization when both LSO-LSO and LSOs-singleton correlations were not significant (none of the duplicates have identical regulatory elements compared to each other) (Table 1), and these genes had the two LSOs located in different heatmap clusters (Fig 2B). Finally, we considered a possible neofunctionalization when LSOs-singleton correlation was significant for one LSO, but not for the other (one copy conserves the same regulatory elements than pre-duplicated singleton while the second copy would acquire or lost some of the elements), and we expected that LSOs appear in different clusters and one of them sharing cluster with the singleton. Interestingly, we did not find any correlation pattern as expected for neofunctionalization; what we found was a significant LSO-LSO correlation but not with the singleton (Table 1), and we observed that most of them had the two LSOs clustering together and the singleton located in a different heatmap cluster (Fig 2B). However, a close analysis of individual genes suggested that it might be a form of neofunctionalization, with the acquisition of new common regulatory elements in both LSO copies. For the majority of the ohnologs, both topology from the cluster analysis and correlation of expression gave the same list of genes related to neofunctionalization, subfunctionalization and redundancy.

**Table 1:** Correlation matrix of gene expression data.

	*LSO/Singleton*	*myod*	*trim63*	*igfbp3*	*tgfb3*	*atf4*	*igf2bp2*	*igf2*	*mef2d*	*pik3ca*	*pip4k2a*	*raf1*	*rictor*	*rragc*	*tgfb1*	*fst*	*grb2*	*eif3j*	*akt2*	*chuk*	*cdc42bpa*
0.75***	*myod1*	0.28*																			
	*myod2*	0.41**																			
0.92***	*trim63a*		0.21																		
	*trim63b*		0.17																		
0.11	*igfbp3a*			0.15																	
	*igfbp3b*			0.03																	
0.16	*tgfb3a*				-0.25																
	*tgfb3b*				-0.13																
0.49**	*atf4a*					-0.24															
	*atf4b*					-0.04															
0.97***	*igf2bp2a*						0.34**														
	*igf2bp2b*						0.28*														
0.53**	*igf2a*							-0.15													
	*igf2b*							-0.11													
0.73***	*mef2da*								0.28*												
	*mef2db*								0.36**												
0.72***	*pik3caa*									0.27*											
	*pik3cab*									0.29*											
0.71***	*pip4k2aa*										0.27*										
	*pip4k2ab*										0.28*										
0.42**	*raf1a*											-0.19									
	*raf1b*											0.36**									
0.64***	*rictora*												-0.08								
	*rictorb*												0.05								
0.48**	*rragca*													0.00							
	*rragcb*													-0.09							
0.41**	*tgfb1a*														0.27*						
	*tgfb1b*														0.23						
0.38**	*fsta*															-0.19					
	*fstb*															-0.23					
0.91***	*grb2a*																-0.06				
	*grb2b*																-0.05				
0.85***	*eif3ja*																	0.35**			
	*eif3jb*																	0.54***			
0.68***	*akt2a*																		-0.28*		
	*akt2b*																		-0.06		
0.63***	*chuka*																			0.08	
	*chukb*																			0.21	
0.79***	*cdc42bpaa*																				0.50***
	*cdc42bpab*																				0.33**

Pearson correlation index between pacu and tilapia LSOs (first column) and singletons (first line) for *myogenic differentiation* (*myod*), *tripartite motif containing 63* (*trim63*), *insulin like growth factor binding protein 3* (*igfbp3*), *transforming growth factor 3* (*tgfb3*), *activating transcription factor 4* (*atf4*), *insulin like growth factor 2 mRNA binding protein 2* (*igf2bp2*), *insulin like growth factor 2* (*igf2*), *myocyte enhancer factor 2D* (*mef2d*), *phosphatidylinositol-4*,*5-bisphosphate 3 kinase catalytic subunit alpha* (*pik3ca*), *phosphatidylinositol-5-phosphate 4 kinase type 2 alpha* (*pip4k2a*), *raf-1 proto-oncogene*, *serine/threonine kinase* (*raf1*), *rptor independent companion of mtor complex 2* (*rictor*), *ras related GTP binding c* (*rragc*), *transforming growth factor beta 1* (*tgfb1*), *follistatin* (*fst*), *growth factor receptor bound protein 2* (*grb2*), *eukaryotic translation initiation factor 3 subunit j* (*eif3j*), *akt serine/threonine kinase 2* (*akt2*), *component of inhibitor of nuclear factor kappa B kinase complex* (*chuk*) and *cdc42 binding protein kinase alpha* (*cdc42bpa*). Significant correlations are indicated with asterisks

*<0.05

**<0.01

***<0.001

If both LSOs-singleton and LSO-LSO correlations were significant (likely have common regulatory elements), it was considered a sign of redundancy (orange). If both LSOs-singleton and LSO-LSO correlations were not significant (likely do not have common regulatory elements), it was considered a sign of subfunctionalization (blue). If LSOs-singleton correlations were not significant (or weak) but LSO-LSO were (both copies likely acquired new common regulatory elements), it was considered a sign of neofunctionalization (green).

**Fig 3:**
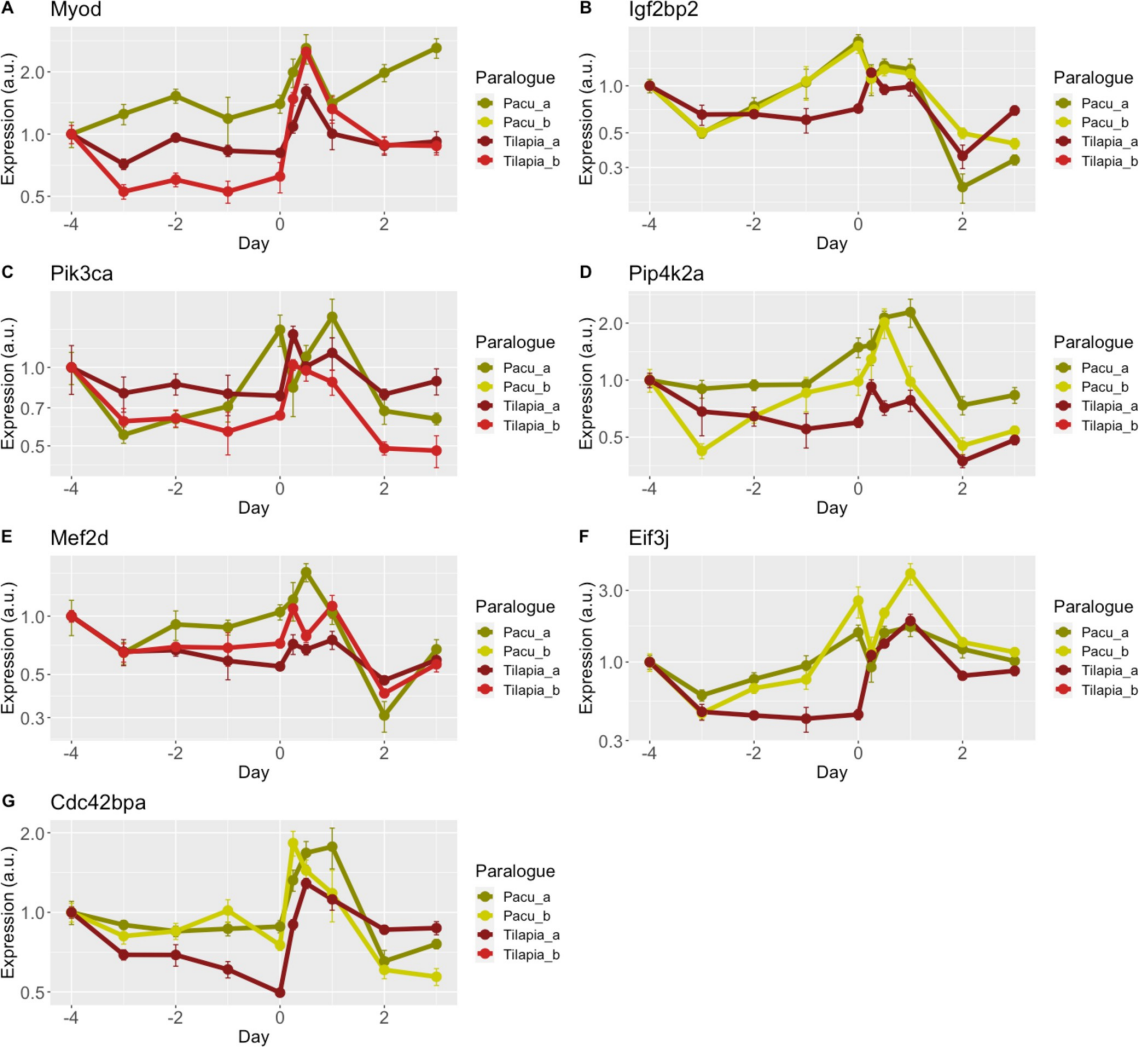
Lineage-specific ohnologs (LSOs) retained by redundancy. Gene duplicates showing signs of redundancy as the main molecular mechanism of retention. LSOs and singletons expression of **(A)** *myogenic differentiation* (*myod*), **(B)** *insulin like growth factor 2 mRNA binding protein 2* (*igf2bp2*), **(C)** *phosphatidylinositol-4*,*5-bisphosphate 3 kinase catalytic subunit alpha* (*pik3ca*), **(D)** *phosphatidylinositol-5-phosphate 4 kinase type 2 alpha* (*pip4k2a*), **(E)** *myocyte enhancer factor 2D* (*mef2d*), **(F)** *eukaryotic translation initiation factor 3 subunit j* (*eif3j*), **(G)** *cdc42 binding protein kinase alpha* (*cdc42bpa*) in pacu (green lines) and tilapia (red lines) skeletal muscle are represented as relative values, Mean±SE (n = 6).

Despite minor variations in their expression, we found 7 genes (*myod*, *igf2bp2*, *pik3ca*, *pip4k2a*, *mef2d*, *eif3j* and *cdc42bpa*; 35% of the total) where transcription profiles, clustering position and correlation values suggested common regulatory elements between LSOs and singletons, a sign of redundancy in response to nutrition (Fig 3A–3G; Table 1). While the general duplication-degeneration-complementation (DCC) paradigm outlines that the majority of ohnologs are retained through neofunctionalization and subfunctionalization [86], it has been suggested that redundancy might account for the retention of ohnologs related to signal transduction, development and metabolic pathways [87, 88]. That has been the case of HOX or MYOD1/MYF5 families in mammals [89, 90]. In the present study, we have found an unusually high proportion of ohnologs with signs of redundancy (35%) that could be explained because LSOs selected in this study were part of metabolic and development networks. However, we cannot rule out that our results are a sign that redundancy might play a bigger role in retention than previously thought.

**Fig 4:**
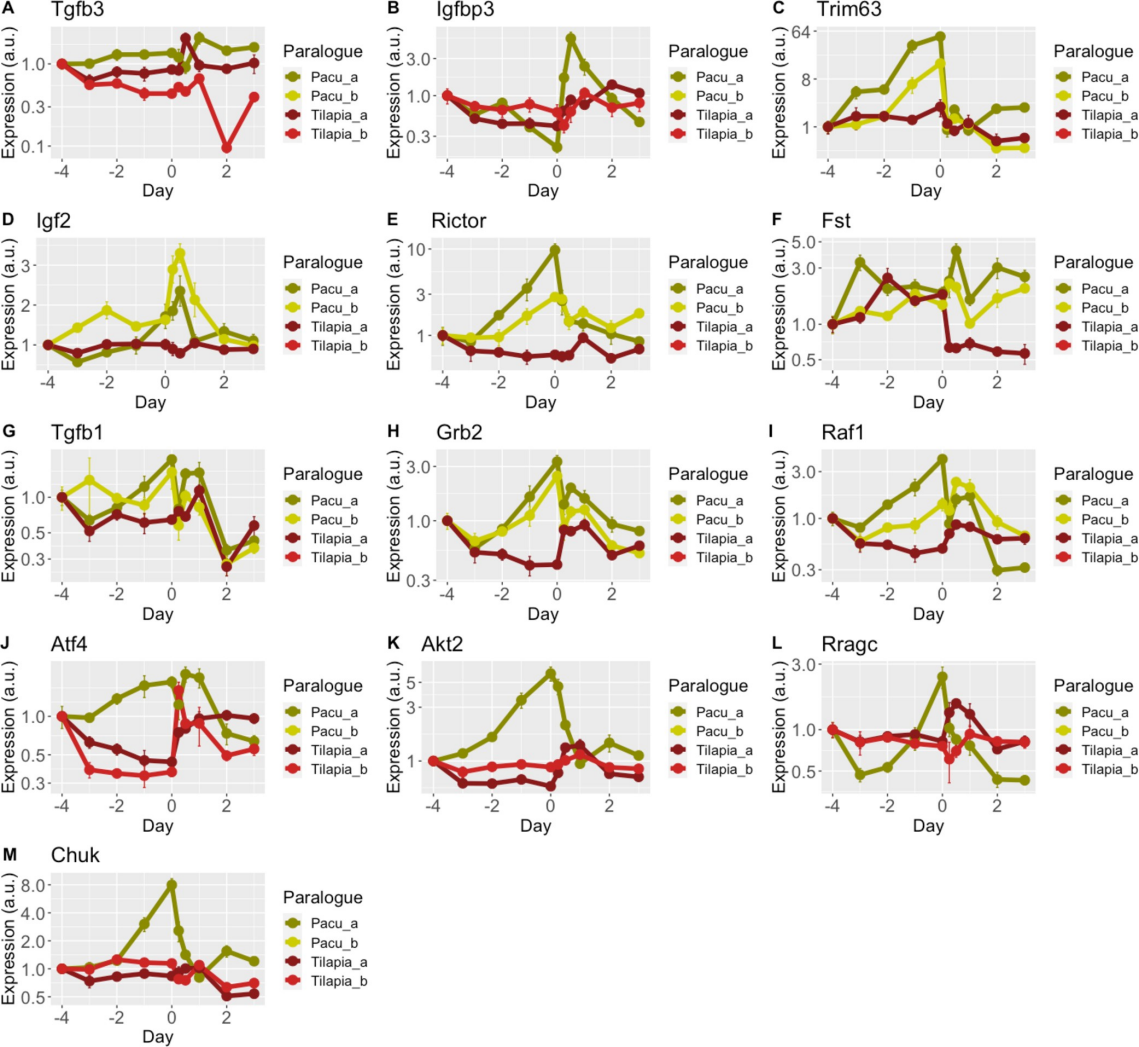
Lineage-specific ohnologs (LSOs) retained by subfunctionalization or neofunctionalization. Gene duplicates showing signs of subfunctionalization and neofunctionalization as the main molecular mechanisms of retention. LSOs and singletons expression of **(A)** *transforming growth factor 3* (*tgfb3*), **(B)** *insulin like growth factor binding protein 3* (*igfbp3*), **(C)** *tripartite motif containing 63* (*trim63*), **(D)** *insulin like growth factor 2* (*igf2*), **(E)** *rptor independent companion of mtor complex 2* (*rictor*), **(F)** *follistatin* (*fst*), **(G)** *transforming growth factor beta 1* (*tgfb1*), **(H)** *growth factor receptor bound protein 2* (*grb2*), **(I)** *raf-1 proto-oncogene*, *serine/threonine kinase* (*raf1*), **(J)** *activating transcription factor 4* (*atf4*), **(K)** *akt serine/threonine kinase 2* (*akt2*), **(L)** *ras related GTP binding c* (*rragc*), **(M)** *component of inhibitor of nuclear factor kappa B kinase complex* (*chuk*) in pacu (green lines) and tilapia (red lines) skeletal muscle are represented as relative values, Mean±SE (n = 6).

The rest of LSOs in the present study (65%) had some significant deviations from the singleton transcription profile that should suggest sub or neofunctionalization (Fig 4). Only *tgfb3* and *igfbp3* (10% of the total) showed an expression profile, clustering topology and correlation values that fit a temporal subfunctionalization showing some complementary expression (Fig 4A and 4B; Table 1). This is a much lower percentage of subfunctionalization that we expected under the DCC paradigm. We have also found a high proportion of LSOs that seemed to have acquired a new regulatory element (both LSOs changed transcription in response to nutrition but not the singleton) (*trim63*, *igf2*, *rictor*, *fst*, *tgfb1*, *grb2* and *raf1*; 35% of the total) (Fig 4C–4I), again a higher proportion than expected under the DCC model. The gain of a new regulatory element by one copy would be an indication of neofunctionalization; however, it is interesting to notice that in all cases both duplicates have acquired the same regulatory element in response to nutrition, not present in the singleton (Fig 4). We have also found four cases (*atf4*, *akt2*, *rragc* and *chuk*; 20% of the total) (Fig 4J–4M) where a possible loss of regulatory elements might have occurred in both copies simultaneously (singleton increase expression but not the LSOs, what we would expect in a process of subfunctionalization). It is quite surprising that both copies of duplicated genes might have acquired or lost the same regulatory elements, especially if we consider that duplicates can have different evolutionary rates [91]. Interestingly, some authors have also suggested that it is possible to acquire new regulatory elements simultaneously in both duplicated genes [92–94]. While this mechanism is not considered as a main molecular mechanism for the acquisition of new functions, our results in the present work might indicate otherwise, at least for relatively recent WGD such as the TWGD. Unfortunately, the lack of an annotated pacu genome do not allow us to identify the gain, loss or retention of regulatory elements between the studied onhologs, and remain to be further confirmed in future studies using accurate and efficient computational and experimental approaches.

We have also to consider that the transcription of LPSs in the present study has been only tested in response to nutrition. Previous works in salmonids have shown that duplicated genes can have similar transcriptomic profiles in response to one stimulus, but completely different in response to another [21]. That could be expected in recently generated duplicated genes such in salmonids [95] that might still go through re-diploidization and common regulatory elements might still present, what would explain our results.

Overall, we showed many duplicated ohnologs with similar transcription profiles in response to pro-growth signals, and that seems to be the case found in other similar studies, where ohnologs related to muscle growth are very similarly regulated by nutrition [21, 59, 96]. Our results suggest that the impact of WGD on the function and regulation of ohnologs related to protein synthesis and myogenesis networks might be smaller than we thought, and redundancy might play a bigger role in the retention of ohnologs, suggesting that evolution tends to conserve ohnolog regulation during muscle development. In addition, some genes showed small, but significant, variations that indicate simultaneous gain or loss of regulatory elements, what also directly impacts the duplicated genes retention after WGD. Our work provides new insights into the diverse teleost fish genome evolution, especially considering genes related to muscle growth pathways differentially preserved between fish species. These evidence can be used as references in further studies of fish muscle growth in an evolutionary perspective, and will also raise awareness of the necessity to better understand the gene regulation of fish onhologs, what would help to select more appropriate markers to study different physiological processes (such as skeletal muscle growth) and could bring improvements in aquaculture programs.

## Supporting information

S1 File List of genes related to protein synthesis, protein degradation and myogenesis networks in skeletal muscle. 238 genes with specific functions in myogenesis, protein synthesis and protein degradation signalling pathways were evaluated to identify lineage-specific ohnologs (LSOs) differently retained between pacus (*Piaractus mesopotamicus*) and Nile tilapias (*Oreochromis niloticus*). Blue colour indicates Ostariophysi-specific ohnologs (retained as two copies in Ostariophysi and one copy in Acanthopterygii), and red colour indicates Acanthopterygii-specific ohnologs (retained as a single copy in Ostariophysi and two copies in Acanthopterygii).
(PDF)

S2 File Teleost LSOs phylogenetic analysis. Phylogenetic reconstruction of the Akt2 (*akt serine/threonine kinase 2*), Atf4 (*activating transcription factor 4*), Cdc42bpa (*cdc42 binding protein kinase alpha*), Chuk (*component of inhibitor of nuclear factor kappa B kinase complex*), Eif3j (*eukaryotic translation initiation factor 3 subunit j*), Fst (*follistatin*), Grb2 (*growth factor receptor bound protein 2*), Igf2 (*insulin like growth factor 2*), Igf2bp2 (*insulin like growth factor 2 mRNA binding protein 2*), Igfbp3 (*insulin like growth factor binding protein 3*), Mef2d (*myocyte enhancer factor 2d*), Myod (*myogenic differentiation*), Pik3ca (*phosphatidylinositol-4*,*5-biphosphate 3-kinase catalytic subunit alpha*), Pip4k2a (*phosphatidylinositol-5-phosphate 4-kinase type 2 alpha*), Raf1 (*raf-1 proto-oncogene*, *serine/threonine kinase*), Rictor (*rptor independent companion of mtor complex 2*), Rragc (*ras-related GTP binding c*), Tgfb1 (*transforming growth factor beta 1*), Tgfb3 (*transforming growth factor beta 3*) and Trim63 (*tripartite motif containing 63*) for different teleost fish species. Bayesian-based phylogenetic trees were constructed from multiple sequence alignments of peptide sequences. Bootstrap-posterior values are indicated on the node of each branch. *Homo sapiens* was used as out-group.
(PDF)

S3 File LSOs accession codes, Real time PCR primer sequences and PCR efficiencies. Primers used for qPCR amplification of the LSOs *akt2*, *atf4*, *cdc42bpa*, *chuk*, *eif3j*, *fst*, *grb2*, *igf2*, *igf2bp2*, *igfbp3*, *mef2d*, *myod*, *pik3ca*, *pip4k2a*, *raf1*, *rictor*, *rragc*, *tgfb1*, *tgfb3* and *trim63*. Primers were also designed for the reference genes *rpl13*, *rpl19* and *ppiaa*, and also for the *fbxo32* to prove the efficacy of fasting-refeeding treatments. Accession code based on European Nucleotide Archive—ENA (accession number PRJEB6656) for pacu [60] and Ensembl Genome Browser 102 database (https://www.ensembl.org) for Nile tilapia.
(PDF)
