# Supplementary material for: An insight on the impact of teleost whole genome duplication on the regulation of the molecular networks controlling skeletal muscle growth
Source: PLoS One. 2021 Jul 22;16(7):e0255006. doi: 10.1371/journal.pone.0255006 (PMC8297816; doi:10.1371/journal.pone.0255006)
Supplement: S1 File — 238 genes with specific functions in myogenesis, protein synthesis and protein degradation signalling pathways were evaluated to identify lineage-specific ohnologs (LSOs) differently retained between pacus (Piaractus mesopotamicus) and Nile tilapias (Oreochromis niloticus). Blue colour indicates Ostariophysi-specific ohnologs (retained as two copies in Ostariophysi and one copy in Acanthopterygii), and red colour indicates Acanthopterygii-specific ohnologs (retained as a single copy in Ostariophysi and two copies in Acanthopterygii). (PDF) [file pone.0255006.s001.pdf]

Protein Synthesis			
Igf1	Akt1	Rraga	
Igf1r	Akt1s1	Rragb	
Igfbp1	Akt2	Rragc	
Igfbp2	Akt3	Rragd	
Igfbp3	Aktip	Lamtor1 (p18)	
Igfbp4	Mtor	Lamtor2 (p14)	
Igfbp5	Rptor	Lamtor3 (Mp1)	
Igfbp6	Rictor	Lamtor4	
Igfbp7	Rps6ka1	Lamtor5	
Igf2	Rps6ka2	Rheb	
Igf2r	Rps6ka3	Rhebl1	
Igf2bp1	Rps6ka4	Tsc1	
Igf2bp2	Rps6ka5	Tsc2	
Igf2bp3	Rps6kb1	Nprl2 (Gator1)	
Pik3ca	Rps6kb2	Nprl3 (Gator1)	
Pik3cb	Eif1b	Depdc5 (Gator1)	
Pik3cd	Eif2s1	Mios (Gator2)	
Pik3cg	Eif2s2	Atp6v0a1	
Pik3c2a	Eif3f	Atp6v0a2	
Pik3c2b	Eif3j	Atp6v0a4	
Pik3c3	Eif5	Atp6v0b	
Pik3r1	Eif5b	Atp6v0c	
Pik3r2	Eif4e	Atp6v0d1	
Pik3r3	Eif4e1	Atp6v0d2	
Pik3r4	Eif4e2	Atp6v0e1	
Pik3r5	Eif4e3	Atp6v0e2	
Pik3ap1	Eif4ebp1	Atp6v1a	
Pik3ip1	Eif4ebp2	Atp6v1b1	V-atpase
Pip4k2a	Eif4ebp3	Atp6v1b2	
	Gh1	Atp6v1c1	
	Ghr	Atp6v1c2	
	Insr	Atp6v1d	
		Atp6v1e1	
		Atp6v1e2	
		Atp6v1f	
		Atp6v1g1	
		Atp6v1g2	
		Atp6v1g3	
		Atp6v1h	

Protein Degradation		
Foxo1	Klf15	
Foxo3	Ddit4 (Redd)	
Foxo4	Fst	
Foxo6	Mstn	
Trim63 (Murf1)	Acvr2a	
Trim55 (Murf2)	Acvr2b	
Trim54 (Murf3)	Smad2	
Fbxo32 (Mafbx)	Smad3	
Fbxo25	Capn1	
Fbxo40	Capn2	
Fbxo11	Cast	
Usp2	Prkaa1	
Usp14	Prkaa2	
Usp19	Prkab1	
Zfand5 (Znf216)	Prkab2	Ampk
Becn1	Prkag1	
Bcl2	Prkag2	
Bnip3	Prkag3	
Bnip3l	Nos1	
Sqstm1 (p62)	Nos2	
Map1lc3 (Lc3)	Junb	
Hsc70	Ppargc1 (Pgc1a)	
Lamp1	Atf4	
Lamp2	March7	
Atg5	Ubr4	
Atg7	Itch	
Atg14	Pja2	
Ulk1	Foxn4	
Ulk2	Fbxl2	
Naf1	Foxk1	
Tnf	Foxk2	
Ikbkb (Ikkb)	Hsp90aa	
Chuk (Ikka)	Hsp90ab	
Nfkb1	Tgfb1	
Nfkb2	Tgfb3	
Tnfsf12 (Tweak)	Unc45a	
Tnfrsf12 (Fn14)	Unc45b	
Traf6		
Nr3c1 (Glucocorticoid receptor)		

Myogenesis	
Myf5	Cav1
Myod	Cav2
Myog	Cav3
Myf6 (Mrf4)	Aox1
Pax3	Gsk3a
Pax7	Gsk3b
Mef2a	Arf6
Mef2b	Nfatc1
Mef2c	Nfatc2
Mef2d	Nfatc3
Akirin1	Ptk2b
Akirin2	Cd9
Vasp	Sp1
Cdh15	Tmem8a
Graf1	Tmem8b
Crkl	Tmem8c
Crkl	Mapk1
Dock1	Mapk3
Dock5	Mapk7
Trio	Mapk8
Triol	Mapk9
Plekho1	Mapk10
Plekho2	Mapk11
Cdc42a	Mapk12
Cdc42bpa	Mapk13
Cdc42bpb	Mapk14 (p38)
Cdc42se1	Mapk15
Cdc42se2	Mapkapk2
Cdc42ep1	
Cdc42ep2	
Cdc42ep3	
Cdc42ep4	
Grb2	
Raf1	

S1 File. List of genes related to protein synthesis, protein degradation and myogenesis networks in skeletal muscle. 238 genes with specific functions in myogenesis, protein synthesis and protein degradation signalling pathways were evaluated to identify lineage-specific ohnologs (LSOs) differently retained between pacus (*Piaractus mesopotamicus*) and Nile tilapias (*Oreochromis niloticus*). Blue colour indicates Ostariophysi-specific ohnologs (retained as two copies in Ostariophysi and one copy in Acanthopterygii), and red colour indicates Acanthopterygii-specific ohnologs (retained as a single copy in Ostariophysi and two copies in Acanthopterygii).
