# Supplementary material for: An insight on the impact of teleost whole genome duplication on the regulation of the molecular networks controlling skeletal muscle growth
Source: PLoS One. 2021 Jul 22;16(7):e0255006. doi: 10.1371/journal.pone.0255006 (PMC8297816; doi:10.1371/journal.pone.0255006)
Supplement: S2 File — Phylogenetic reconstruction of the Akt2 (akt serine/threonine kinase 2), Atf4 (activating transcription factor 4), Cdc42bpa (cdc42 binding protein kinase alpha), Chuk (component of inhibitor of nuclear factor kappa B kinase complex), Eif3j (eukaryotic translation initiation factor 3 subunit j), Fst (follistatin), Grb2 (growth factor receptor bound protein 2), Igf2 (insulin like growth factor 2), Igf2bp2 (insulin like growth factor 2 mRNA binding protein 2), Igfbp3 (insulin like growth factor binding protein 3), Mef2d (myocyte enhancer factor 2d), Myod (myogenic differentiation), Pik3ca (phosphatidylinositol-4,5-biphosphate 3-kinase catalytic subunit alpha), Pip4k2a (phosphatidylinositol-5-phosphate 4-kinase type 2 alpha), Raf1 (raf-1 proto-oncogene, serine/threonine kinase), Rictor (rptor independent companion of mtor complex 2), Rragc (ras-related GTP binding c), Tgfb1 (transforming growth factor beta 1), Tgfb3 (transforming growth factor beta 3) and Trim63 (tripartite motif containing 63) for different teleost fish species. Bayesian-based phylogenetic trees were constructed from multiple sequence alignments of peptide sequences. Bootstrap-posterior values are indicated on the node of each branch. Homo sapiens was used as out-group. (PDF) [file pone.0255006.s002.pdf]

S2 File. Teleost LSOs phylogenetic analysis. Phylogenetic reconstruction of the Akt2 (*akt serine/threonine kinase 2*), Atf4 (*activating transcription factor 4*), Cdc42bpa (*cdc42 binding protein kinase alpha*), Chuk (*component of inhibitor of nuclear factor kappa B kinase complex*), Eif3j (*eukaryotic translation initiation factor 3 subunit j*), Fst (*follistatin*), Grb2 (*growth factor receptor bound protein 2*), Igf2 (*insulin like growth factor 2*), Igf2bp2 (*insulin like growth factor 2 mRNA binding protein 2*), Igfbp3 (*insulin like growth factor binding protein 3*), Mef2d (*myocyte enhancer factor 2d*), Myod (*myogenic differentiation*), Pik3ca (*phosphatidylinositol-4,5-biphosphate 3-kinase catalytic subunit alpha*), Pip4k2a (*phosphatidylinositol-5-phosphate 4-kinase type 2 alpha*), Raf1 (*raf-1 proto-oncogene, serine/threonine kinase*), Rictor (*rptor independent companion of mtor complex 2*), Rragc (*ras-related GTP binding c*), Tgfb1 (*transforming growth factor beta 1*), Tgfb3 (*transforming growth factor beta 3*) and Trim63 (*tripartite motif containing 63*) for different teleost fish species. Bayesian-based phylogenetic trees were constructed from multiple sequence alignments of peptide sequences. Bootstrap-posterior values are indicated on the node of each branch. *Homo sapiens* was used as out-group.

Akt2

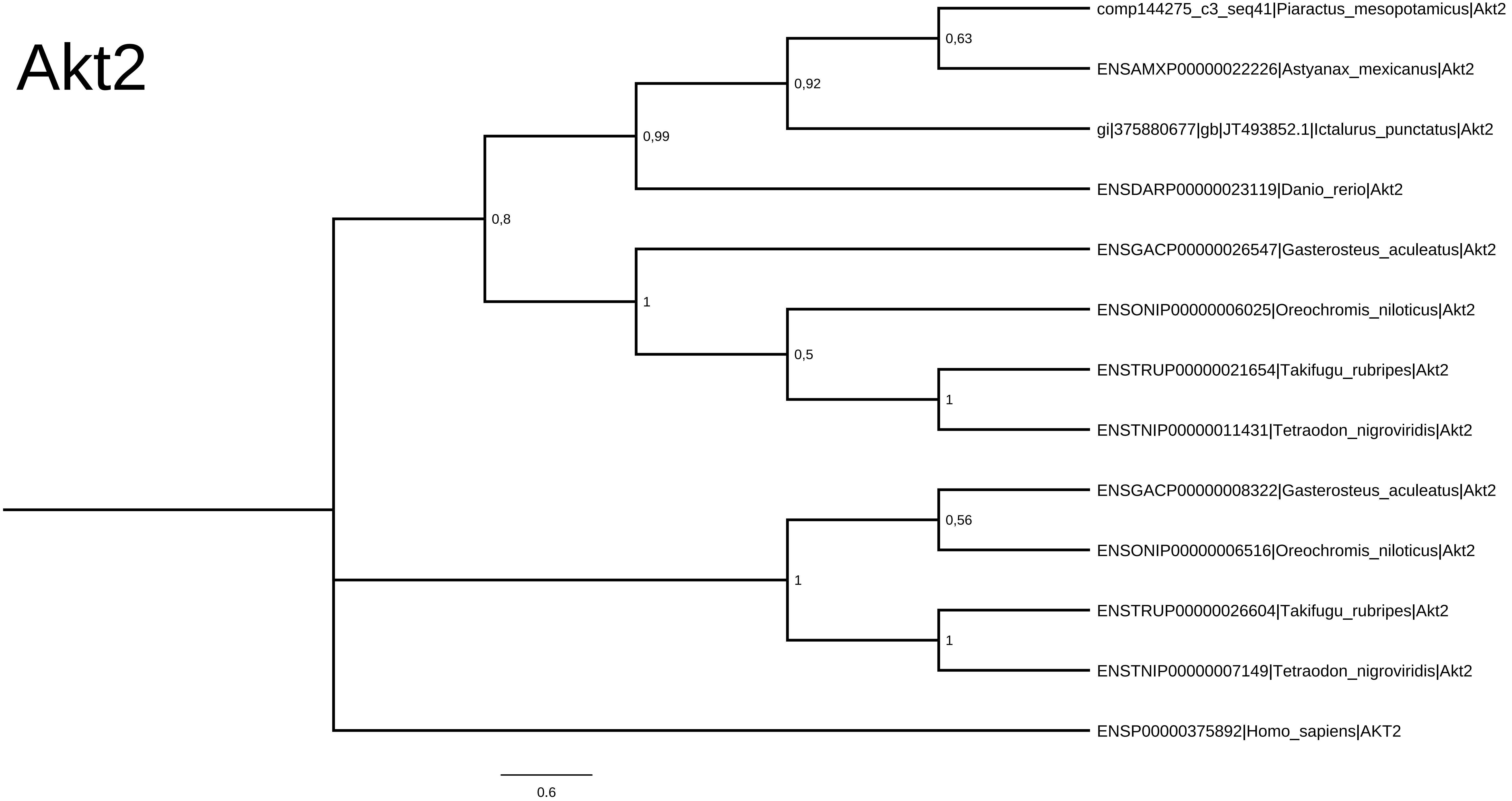
comp144275_c3_seq41|Piaractus_mesopotamicus|Akt2
ENSAMXP00000022226|Astyanax_mexicanus|Akt2
gi|375880677|gb|JT493852.1|Ictalurus_punctatus|Akt2
ENSDARP00000023119|Danio_rerio|Akt2
ENSGACP00000026547|Gasterosteus_aculeatus|Akt2
ENSONIP00000006025|Oreochromis_niloticus|Akt2
ENSTRUP00000021654|Takifugu_rubripes|Akt2
ENSTNIP00000011431|Tetraodon_nigroviridis|Akt2
ENSGACP00000008322|Gasterosteus_aculeatus|Akt2
ENSONIP00000006516|Oreochromis_niloticus|Akt2
ENSTRUP00000026604|Takifugu_rubripes|Akt2
ENSTNIP00000007149|Tetraodon_nigroviridis|Akt2
ENSP00000375892|Homo_sapiens|AKT2
0,63
0,92
0,99
0,8
1
0,5
1
0,56
1
1
0.6

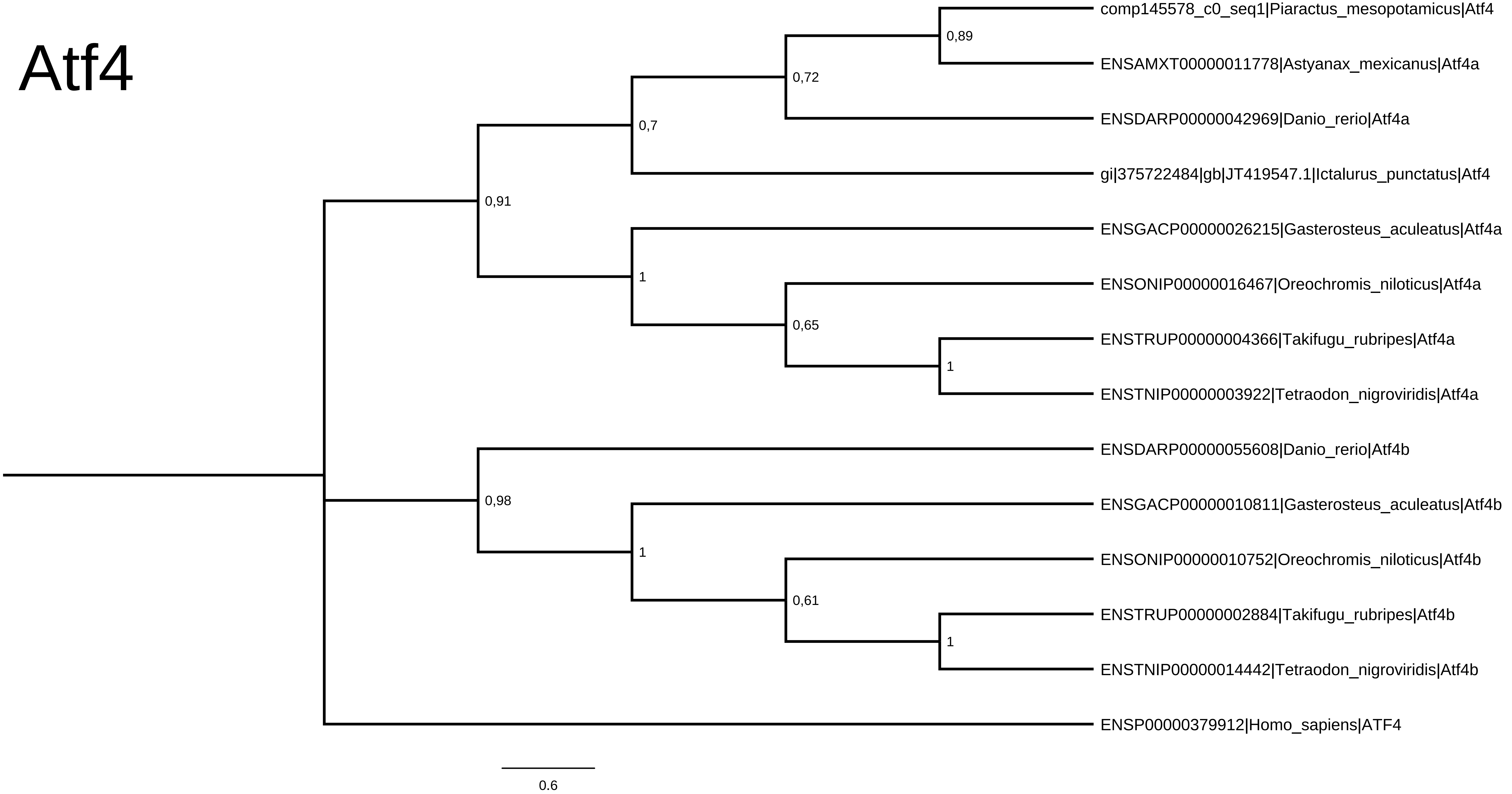
Atf4
comp145578_c0_seq1|Piaractus_mesopotamicus|Atf4
ENSAMXT00000011778|Astyanax_mexicanus|Atf4a
ENSDARP00000042969|Danio_rerio|Atf4a
gi|375722484|gb|JT419547.1|Ictalurus_punctatus|Atf4
ENSGACP00000026215|Gasterosteus_aculeatus|Atf4a
ENSONIP00000016467|Oreochromis_niloticus|Atf4a
ENSTRUP00000004366|Takifugu_rubripes|Atf4a
ENSTNIP00000003922|Tetraodon_nigroviridis|Atf4a
ENSDARP00000055608|Danio_rerio|Atf4b
ENSGACP00000010811|Gasterosteus_aculeatus|Atf4b
ENSONIP00000010752|Oreochromis_niloticus|Atf4b
ENSTRUP00000002884|Takifugu_rubripes|Atf4b
ENSTNIP00000014442|Tetraodon_nigroviridis|Atf4b
ENSP00000379912|Homo_sapiens|ATF4
0,89
0,72
0,7
0,91
1
0,65
1
0,98
1
0,61
1
0.6

Cdc42bpa

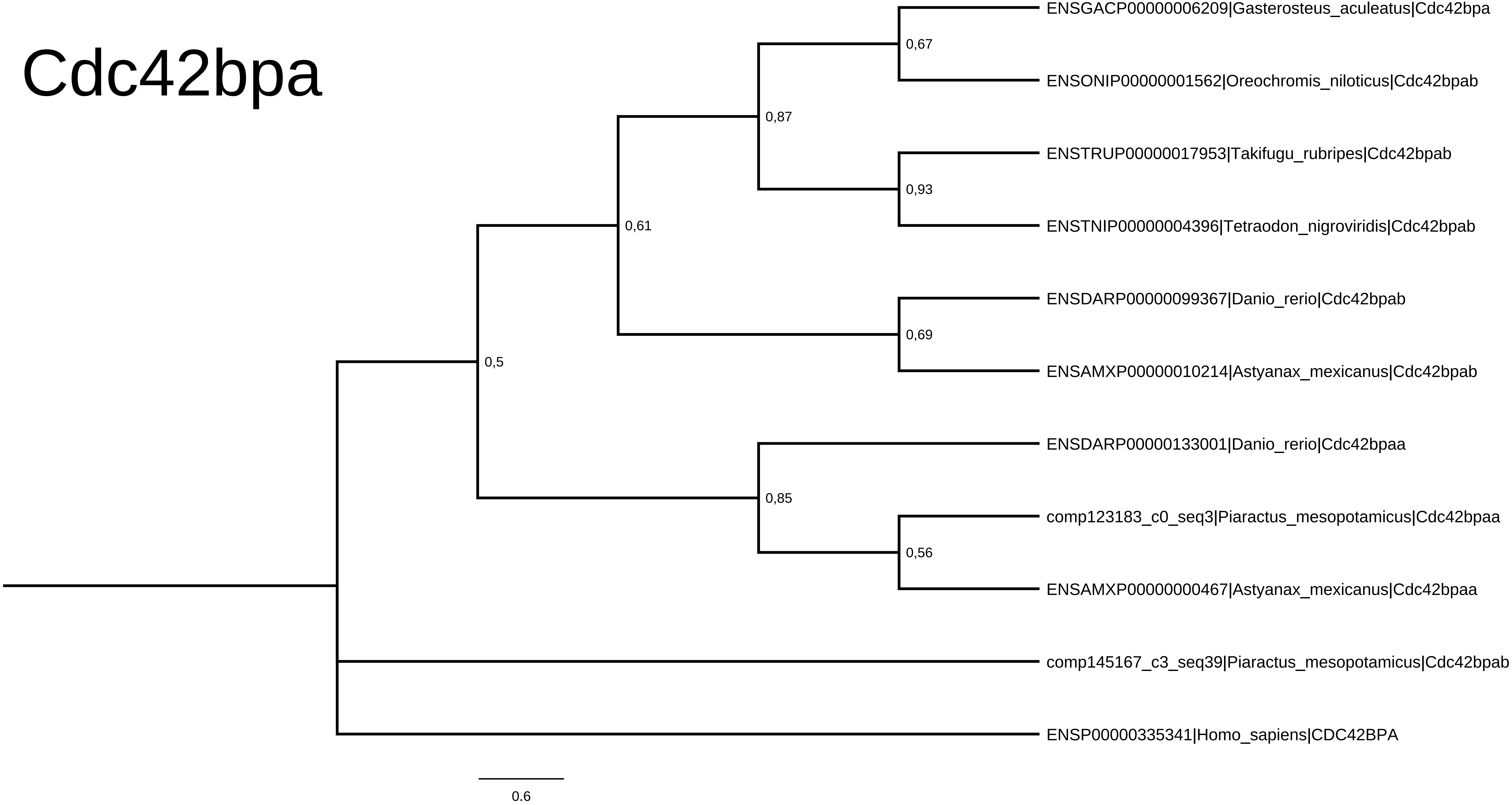

ENSGACP00000006209|Gasterosteus_aculeatus|Cdc42bpa
0,67
ENSONIP00000001562|Oreochromis_niloticus|Cdc42bpab
0,87
ENSTRUP00000017953|Takifugu_rubripes|Cdc42bpab
0,93
ENSTNIP00000004396|Tetraodon_nigroviridis|Cdc42bpab
0,61
ENSDARP00000099367|Danio_rerio|Cdc42bpab
0,69
ENSAMXP00000010214|Astyanax_mexicanus|Cdc42bpab
0,5
ENSDARP00000133001|Danio_rerio|Cdc42bpaa
0,85
comp123183_c0_seq3|Piaractus_mesopotamicus|Cdc42bpaa
0,56
ENSAMXP00000000467|Astyanax_mexicanus|Cdc42bpaa
comp145167_c3_seq39|Piaractus_mesopotamicus|Cdc42bpab
ENSP00000335341|Homo_sapiens|CDC42BPA
0.6

Chuk

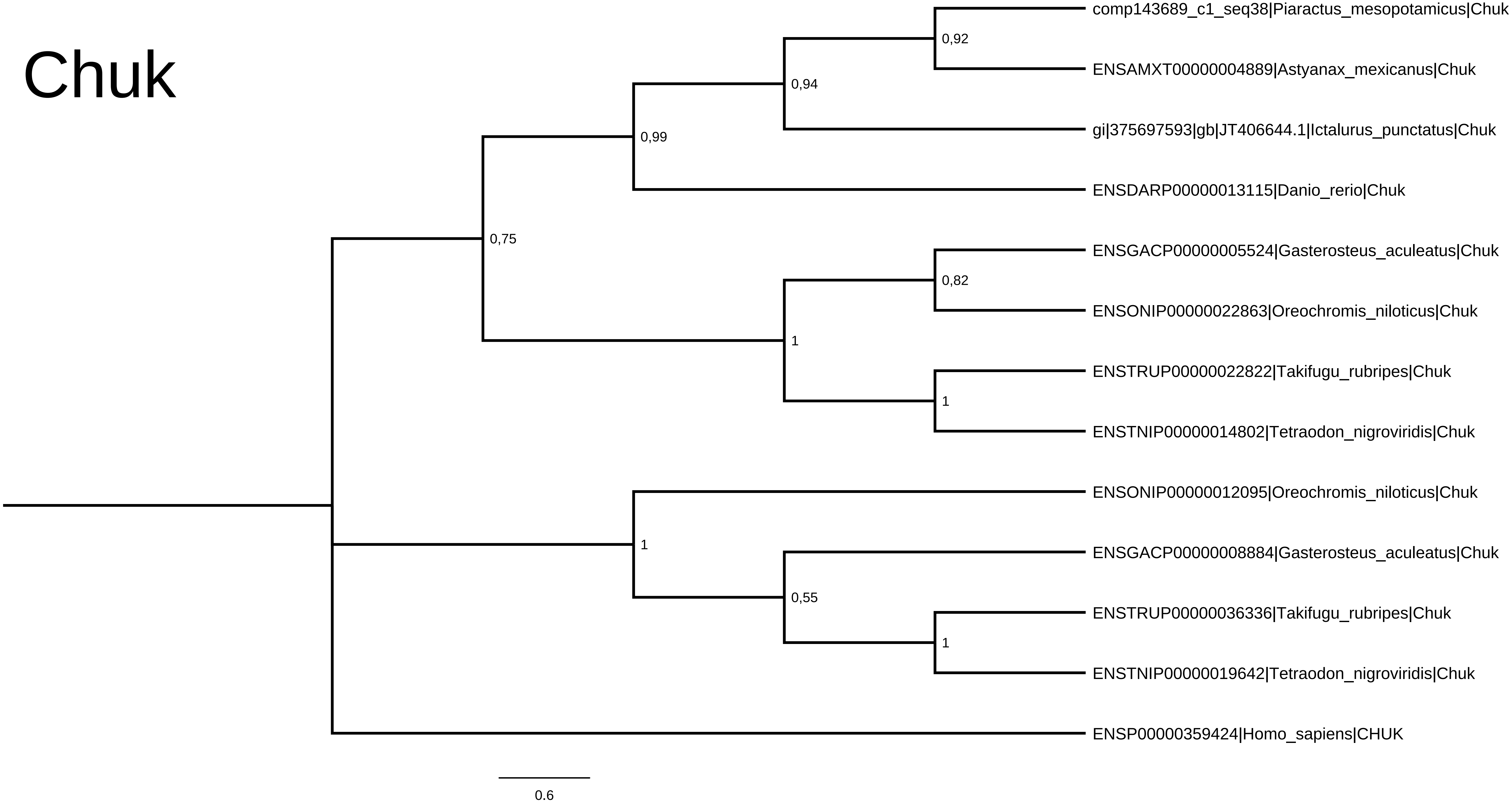

comp143689_c1_seq38|Piaractus_mesopotamicus|Chuk
0,92
ENSAMXT00000004889|Astyanax_mexicanus|Chuk
0,94
gi|375697593|gb|JT406644.1|Ictalurus_punctatus|Chuk
0,99
ENSDARP00000013115|Danio_rerio|Chuk
0,75
ENSGACP00000005524|Gasterosteus_aculeatus|Chuk
0,82
ENSONIP00000022863|Oreochromis_niloticus|Chuk
1
ENSTRUP00000022822|Takifugu_rubripes|Chuk
1
ENSTNIP00000014802|Tetraodon_nigroviridis|Chuk
ENSONIP00000012095|Oreochromis_niloticus|Chuk
1
ENSGACP00000008884|Gasterosteus_aculeatus|Chuk
0,55
ENSTRUP00000036336|Takifugu_rubripes|Chuk
1
ENSTNIP00000019642|Tetraodon_nigroviridis|Chuk
ENSP00000359424|Homo_sapiens|CHUK
0.6

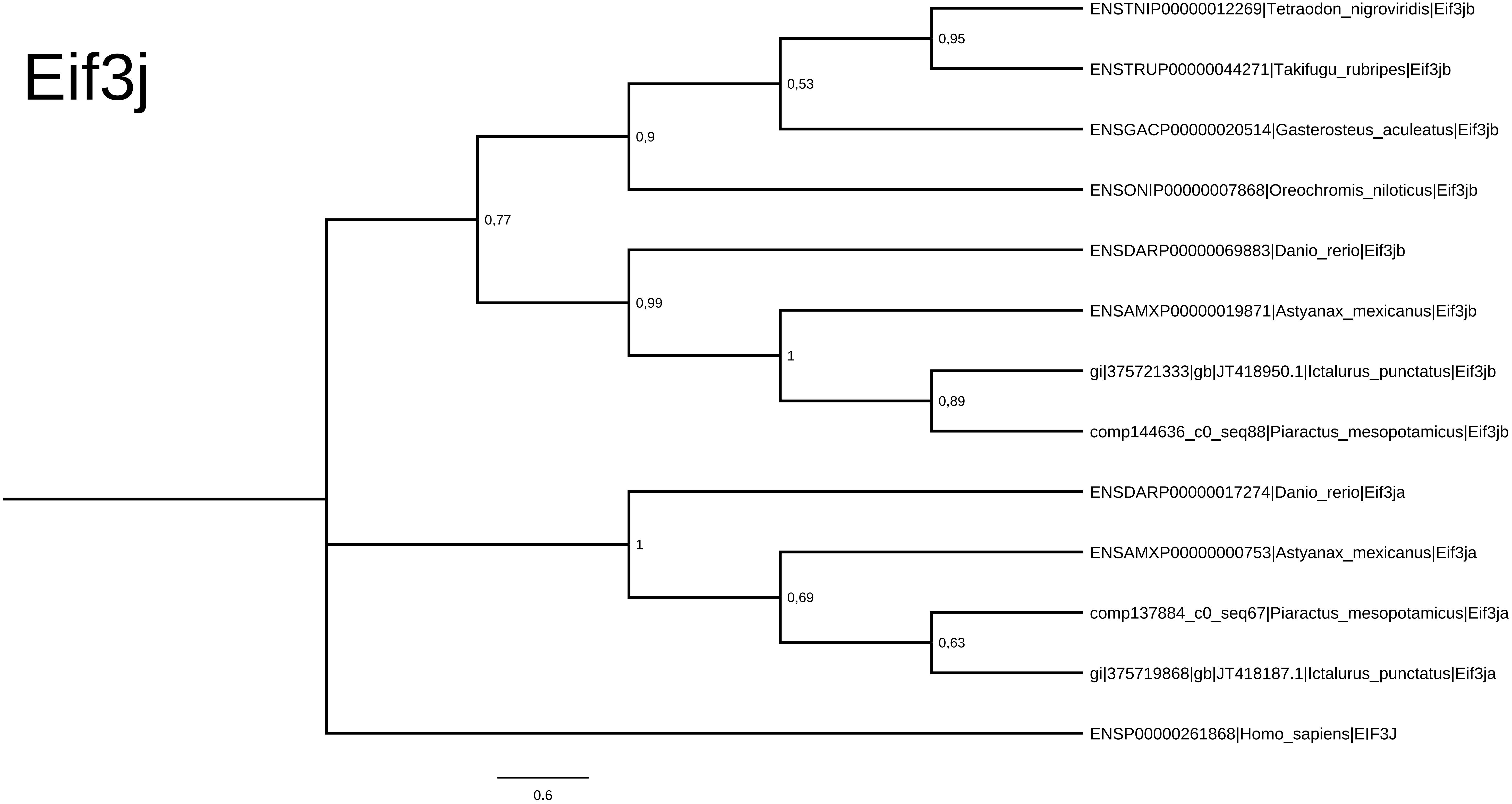
Eif3j
ENSTNIP00000012269|Tetraodon_nigroviridis|Eif3jb
ENSTRUP00000044271|Takifugu_rubripes|Eif3jb
ENSGACP00000020514|Gasterosteus_aculeatus|Eif3jb
ENSONIP00000007868|Oreochromis_niloticus|Eif3jb
ENSDARP00000069883|Danio_rerio|Eif3jb
ENSAMXP00000019871|Astyanax_mexicanus|Eif3jb
gi|375721333|gb|JT418950.1|Ictalurus_punctatus|Eif3jb
comp144636_c0_seq88|Piaractus_mesopotamicus|Eif3jb
ENSDARP00000017274|Danio_rerio|Eif3ja
ENSAMXP00000000753|Astyanax_mexicanus|Eif3ja
comp137884_c0_seq67|Piaractus_mesopotamicus|Eif3ja
gi|375719868|gb|JT418187.1|Ictalurus_punctatus|Eif3ja
ENSP00000261868|Homo_sapiens|EIF3J
0,95
0,53
0,9
0,77
0,99
1
0,89
1
0,69
0,63
0.6

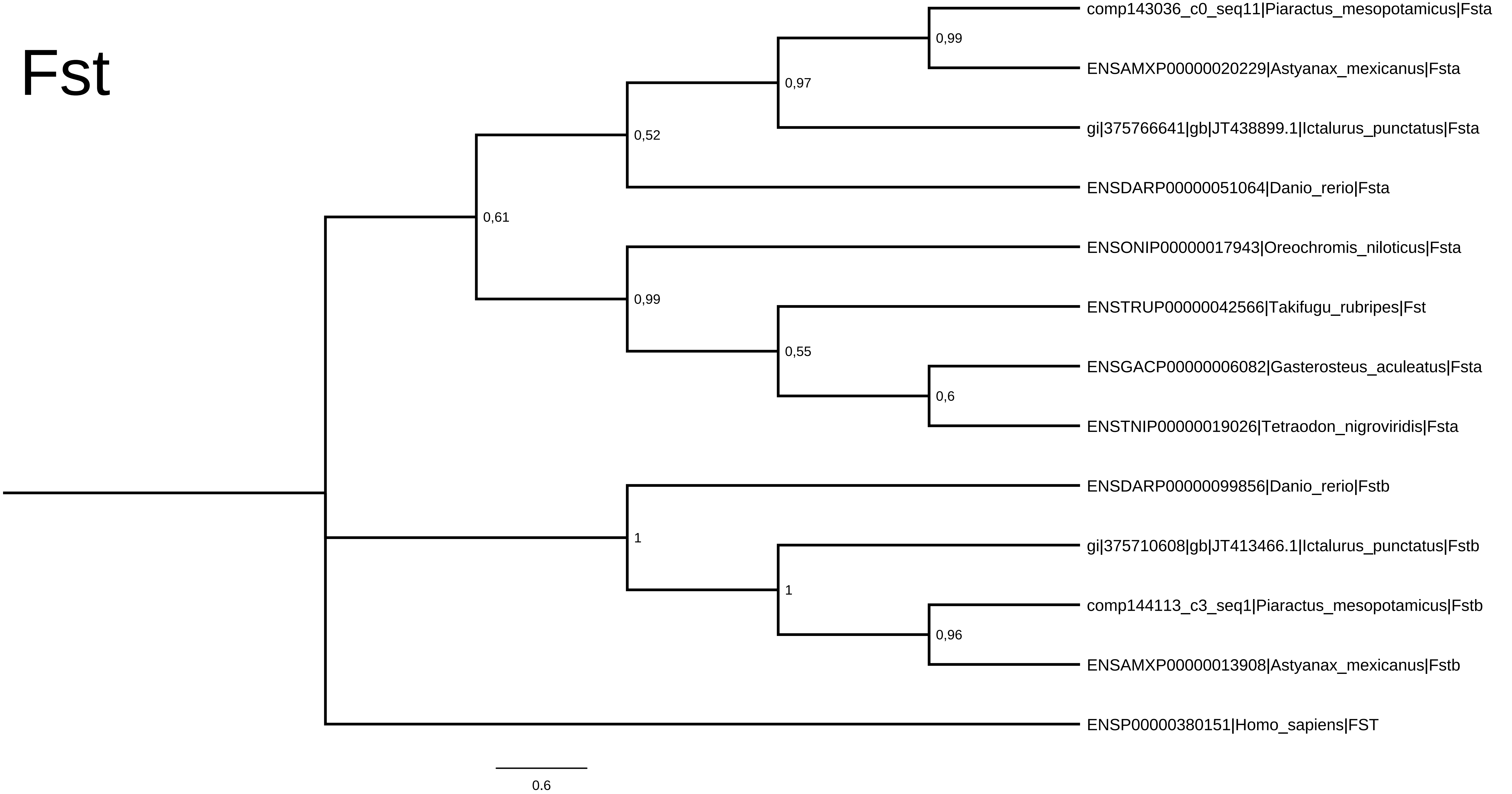
Fst
comp143036_c0_seq11|Piaractus_mesopotamicus|Fsta
0,99
ENSAMXP00000020229|Astyanax_mexicanus|Fsta
0,97
gi|375766641|gb|JT438899.1|Ictalurus_punctatus|Fsta
0,52
ENSDARP00000051064|Danio_rerio|Fsta
0,61
ENSONIP00000017943|Oreochromis_niloticus|Fsta
0,99
ENSTRUP00000042566|Takifugu_rubripes|Fst
0,55
ENSGACP00000006082|Gasterosteus_aculeatus|Fsta
0,6
ENSTNIP00000019026|Tetraodon_nigroviridis|Fsta
ENSDARP00000099856|Danio_rerio|Fstb
1
gi|375710608|gb|JT413466.1|Ictalurus_punctatus|Fstb
1
comp144113_c3_seq1|Piaractus_mesopotamicus|Fstb
0,96
ENSAMXP00000013908|Astyanax_mexicanus|Fstb
ENSP00000380151|Homo_sapiens|FST
0.6

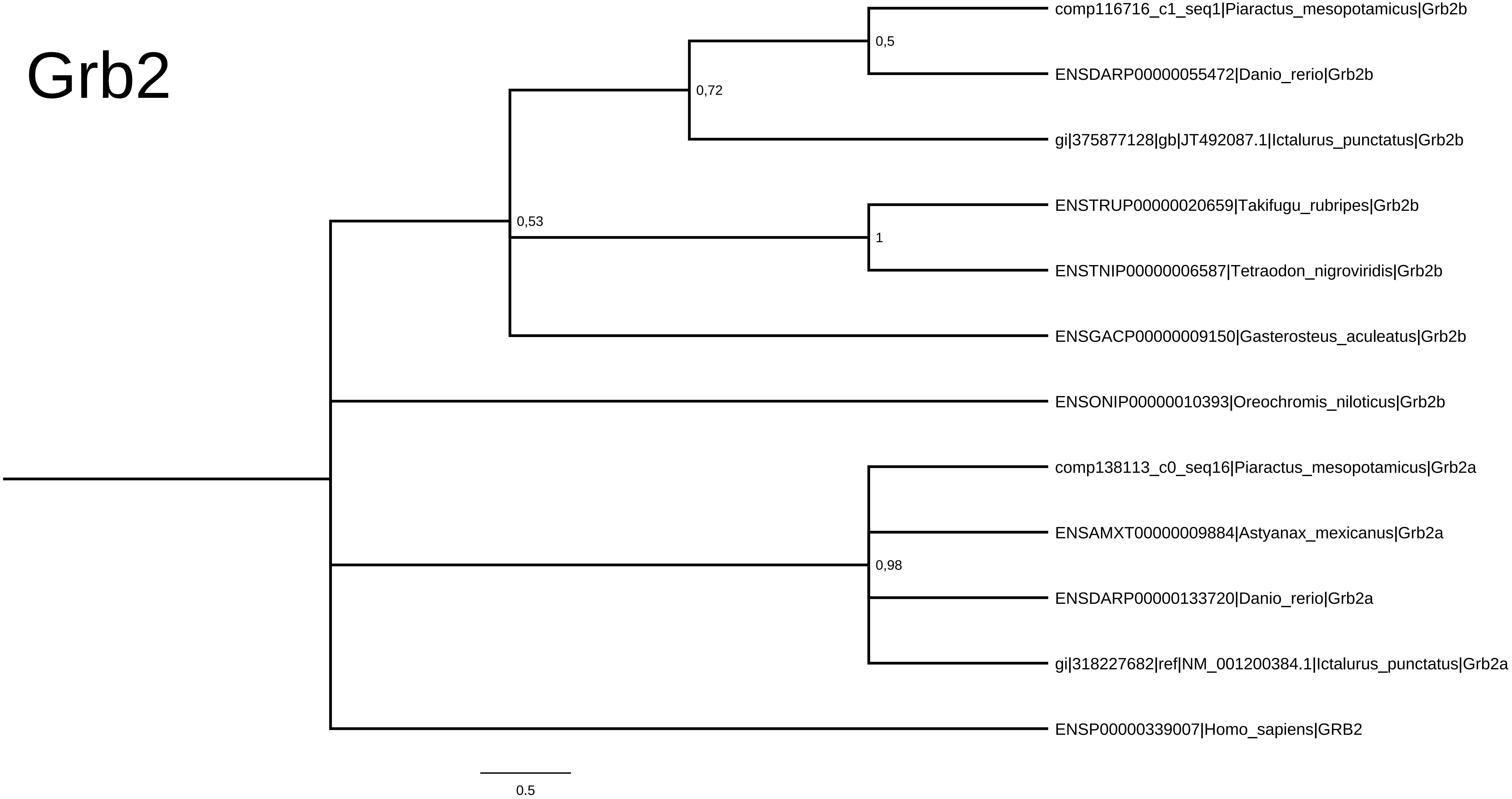
Grb2
comp116716_c1_seq1|Piaractus_mesopotamicus|Grb2b
0,5
ENSDARP00000055472|Danio_rerio|Grb2b
0,72
gi|375877128|gb|JT492087.1|Ictalurus_punctatus|Grb2b
ENSTRUP00000020659|Takifugu_rubripes|Grb2b
0,53
1
ENSTNIP00000006587|Tetraodon_nigroviridis|Grb2b
ENSGACP00000009150|Gasterosteus_aculeatus|Grb2b
ENSONIP00000010393|Oreochromis_niloticus|Grb2b
comp138113_c0_seq16|Piaractus_mesopotamicus|Grb2a
ENSAMXT00000009884|Astyanax_mexicanus|Grb2a
0,98
ENSDARP00000133720|Danio_rerio|Grb2a
gi|318227682|ref|NM_001200384.1|Ictalurus_punctatus|Grb2a
ENSP00000339007|Homo_sapiens|GRB2
0.5

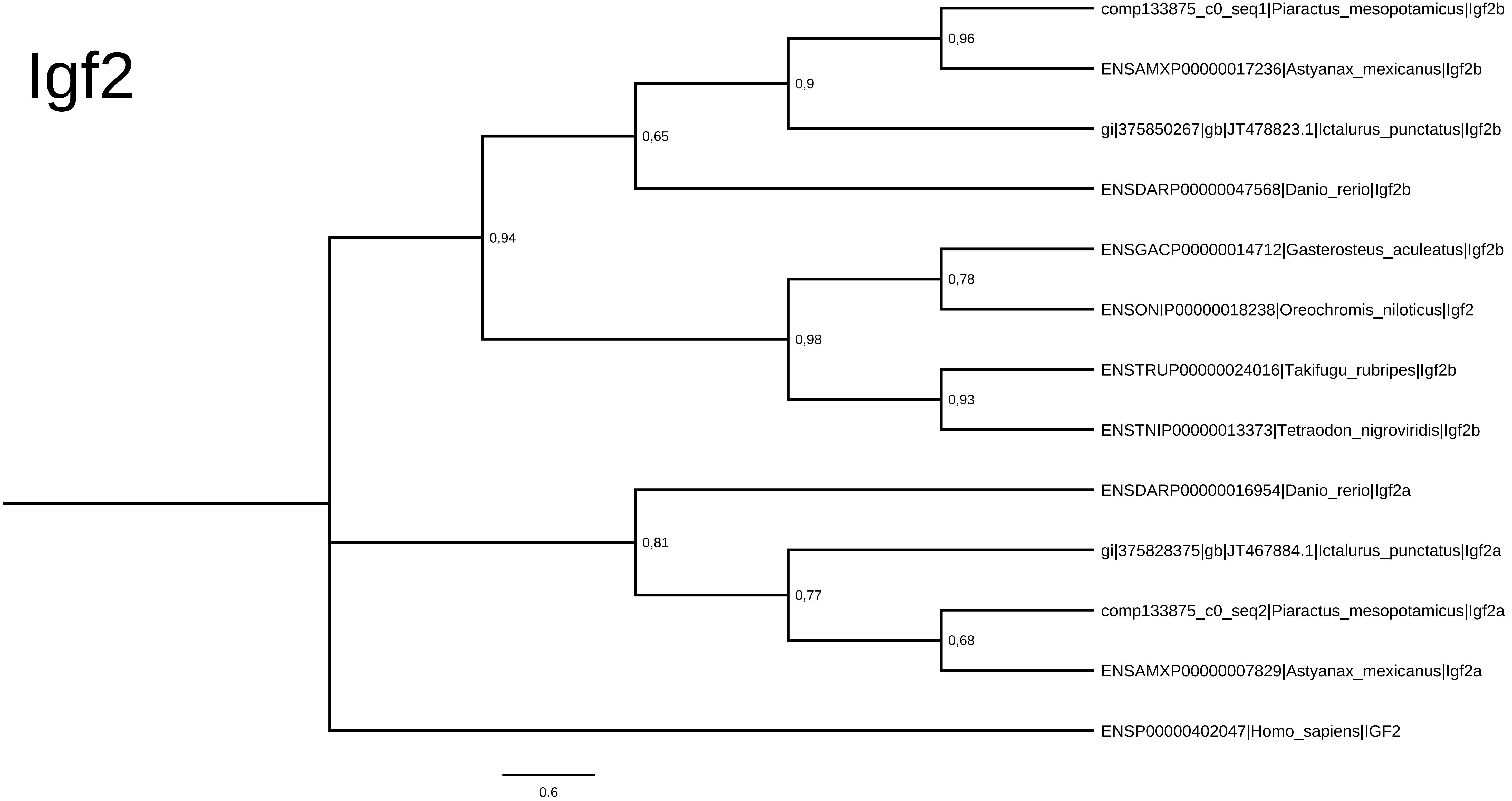
Igf2
comp133875_c0_seq1|Piaractus_mesopotamicus|Igf2b
ENSAMXP00000017236|Astyanax_mexicanus|Igf2b
gi|375850267|gb|JT478823.1|Ictalurus_punctatus|Igf2b
ENSDARP00000047568|Danio_rerio|Igf2b
ENSGACP00000014712|Gasterosteus_aculeatus|Igf2b
ENSONIP00000018238|Oreochromis_niloticus|Igf2
ENSTRUP00000024016|Takifugu_rubripes|Igf2b
ENSTNIP00000013373|Tetraodon_nigroviridis|Igf2b
ENSDARP00000016954|Danio_rerio|Igf2a
gi|375828375|gb|JT467884.1|Ictalurus_punctatus|Igf2a
comp133875_c0_seq2|Piaractus_mesopotamicus|Igf2a
ENSAMXP00000007829|Astyanax_mexicanus|Igf2a
ENSP00000402047|Homo_sapiens|IGF2
0,96
0,9
0,65
0,94
0,78
0,98
0,93
0,81
0,77
0,68
0.6

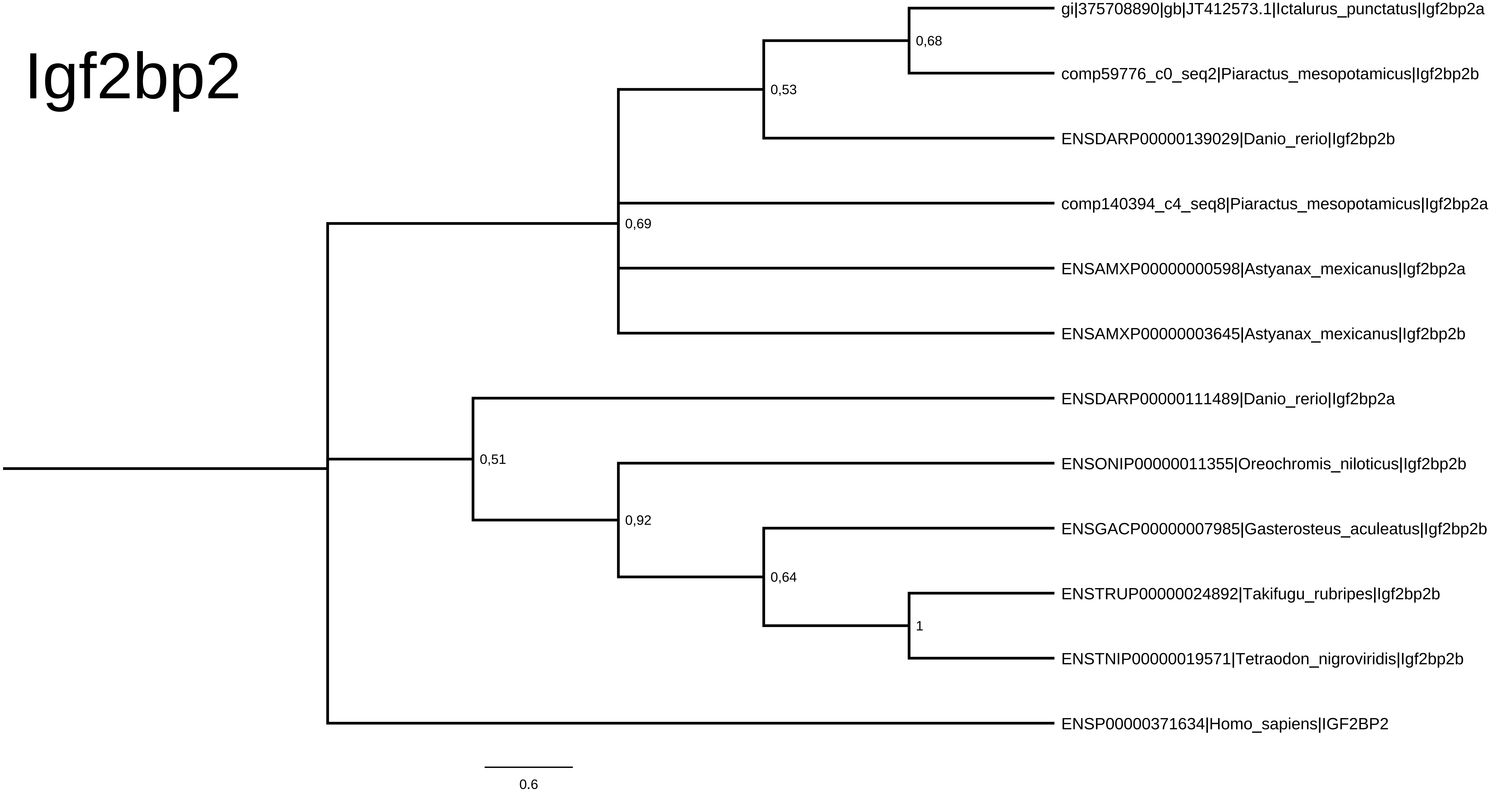

Igf2bp2
gi|375708890|gb|JT412573.1|Ictalurus_punctatus|Igf2bp2a
0,68
comp59776_c0_seq2|Piaractus_mesopotamicus|Igf2bp2b
0,53
ENSDARP00000139029|Danio_rerio|Igf2bp2b
comp140394_c4_seq8|Piaractus_mesopotamicus|Igf2bp2a
0,69
ENSAMXP00000000598|Astyanax_mexicanus|Igf2bp2a
ENSAMXP00000003645|Astyanax_mexicanus|Igf2bp2b
ENSDARP00000111489|Danio_rerio|Igf2bp2a
0,51
ENSONIP00000011355|Oreochromis_niloticus|Igf2bp2b
0,92
ENSGACP00000007985|Gasterosteus_aculeatus|Igf2bp2b
0,64
ENSTRUP00000024892|Takifugu_rubripes|Igf2bp2b
1
ENSTNIP00000019571|Tetraodon_nigroviridis|Igf2bp2b
ENSP00000371634|Homo_sapiens|IGF2BP2
0.6

Igfbp3

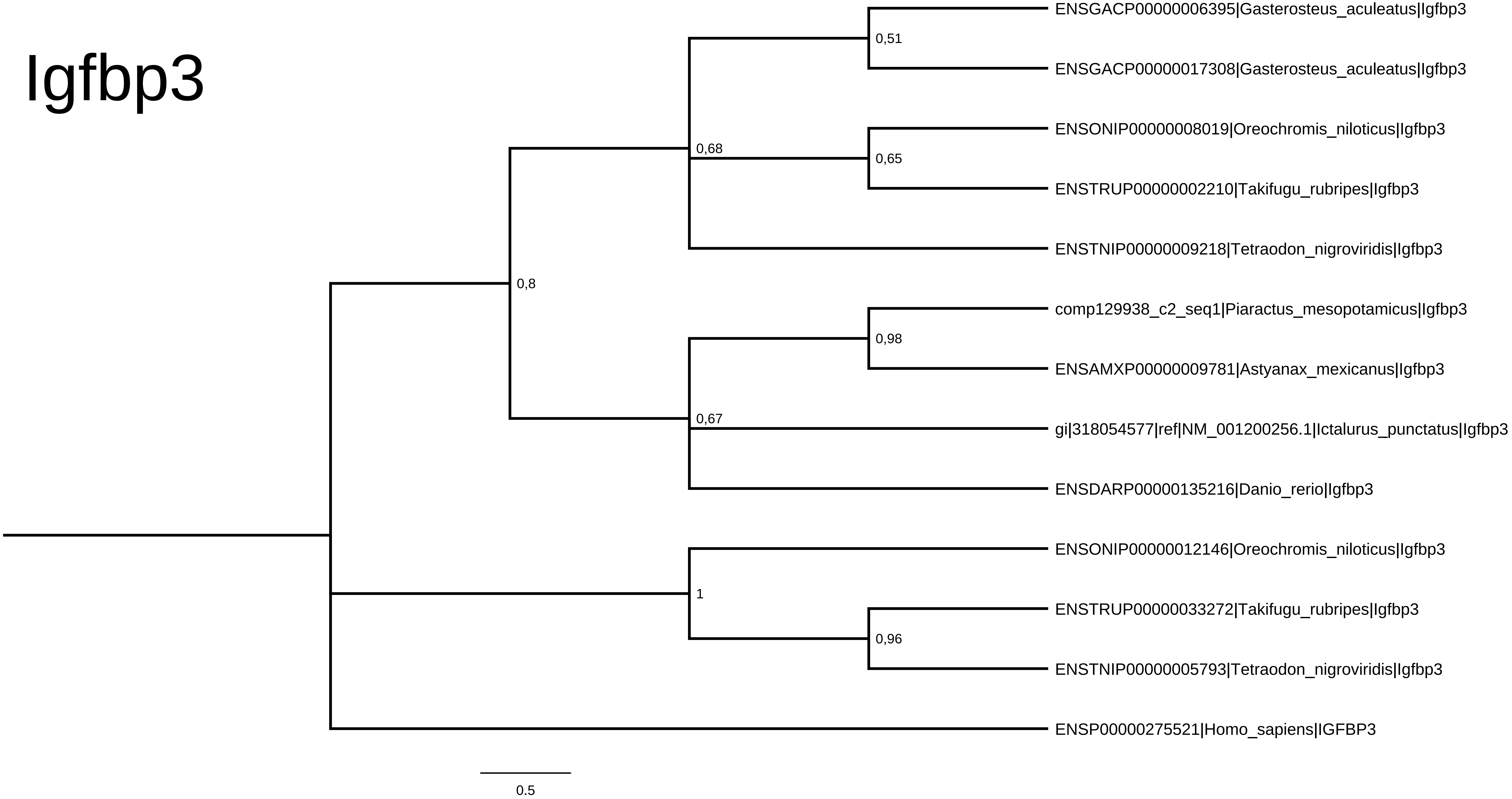
ENSGACP00000006395|Gasterosteus_aculeatus|Igfbp3
0,51
ENSGACP00000017308|Gasterosteus_aculeatus|Igfbp3
ENSONIP00000008019|Oreochromis_niloticus|Igfbp3
0,68
0,65
ENSTRUP00000002210|Takifugu_rubripes|Igfbp3
ENSTNIP00000009218|Tetraodon_nigroviridis|Igfbp3
0,8
comp129938_c2_seq1|Piaractus_mesopotamicus|Igfbp3
0,98
ENSAMXP00000009781|Astyanax_mexicanus|Igfbp3
0,67
gi|318054577|ref|NM_001200256.1|Ictalurus_punctatus|Igfbp3
ENSDARP00000135216|Danio_rerio|Igfbp3
ENSONIP00000012146|Oreochromis_niloticus|Igfbp3
1
ENSTRUP00000033272|Takifugu_rubripes|Igfbp3
0,96
ENSTNIP00000005793|Tetraodon_nigroviridis|Igfbp3
ENSP00000275521|Homo_sapiens|IGFBP3
0.5

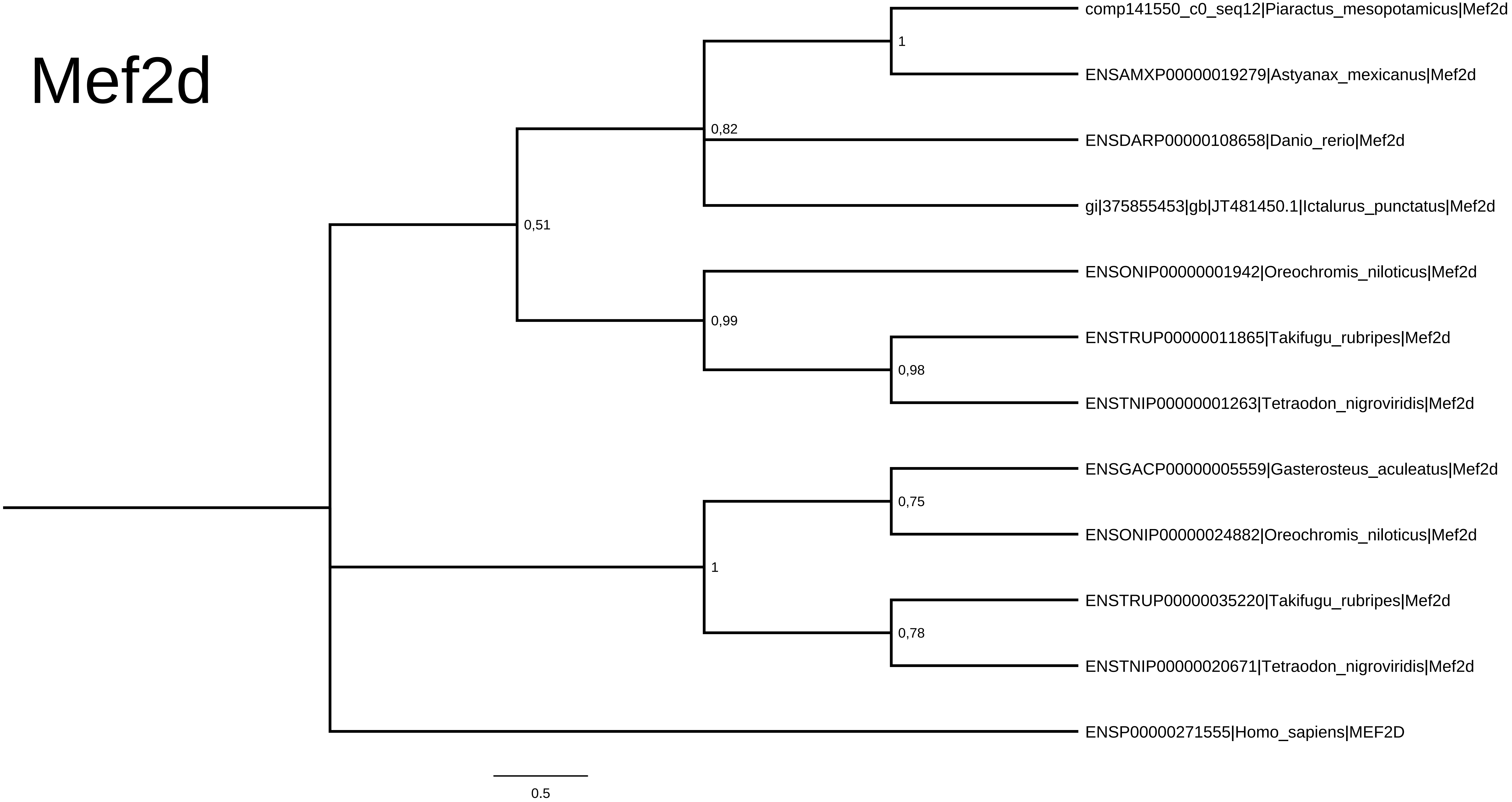

Mef2d
comp141550_c0_seq12|Piaractus_mesopotamicus|Mef2d
ENSAMXP00000019279|Astyanax_mexicanus|Mef2d
ENSDARP00000108658|Danio_rerio|Mef2d
gi|375855453|gb|JT481450.1|Ictalurus_punctatus|Mef2d
ENSONIP00000001942|Oreochromis_niloticus|Mef2d
ENSTRUP00000011865|Takifugu_rubripes|Mef2d
ENSTNIP00000001263|Tetraodon_nigroviridis|Mef2d
ENSGACP00000005559|Gasterosteus_aculeatus|Mef2d
ENSONIP00000024882|Oreochromis_niloticus|Mef2d
ENSTRUP00000035220|Takifugu_rubripes|Mef2d
ENSTNIP00000020671|Tetraodon_nigroviridis|Mef2d
ENSP00000271555|Homo_sapiens|MEF2D
1
0,82
0,51
0,99
0,98
0,75
1
0,78
0.5

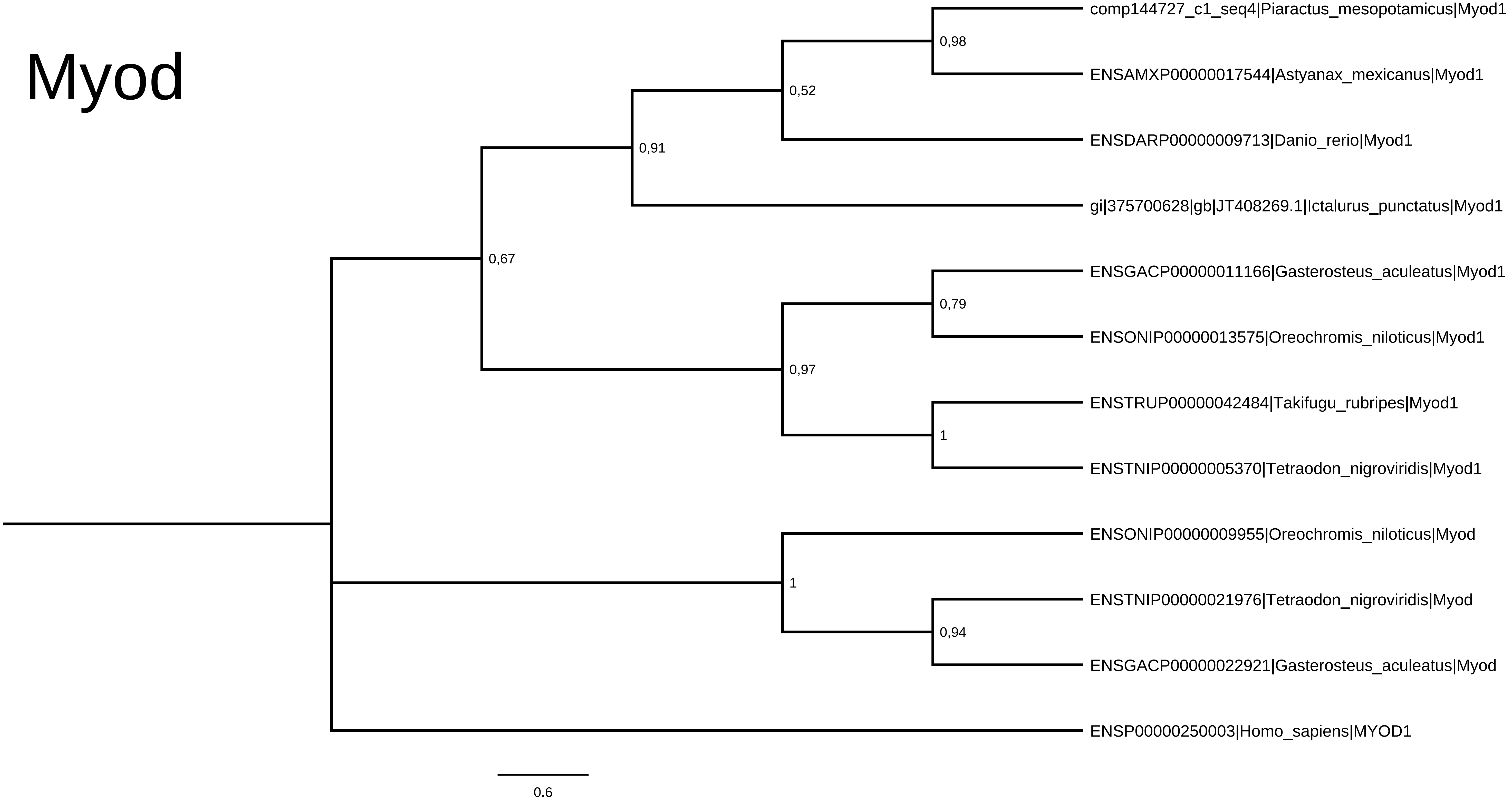

Myod
comp144727_c1_seq4|Piaractus_mesopotamicus|Myod1
ENSAMXP00000017544|Astyanax_mexicanus|Myod1
ENSDARP00000009713|Danio_rerio|Myod1
gi|375700628|gb|JT408269.1|Ictalurus_punctatus|Myod1
ENSGACP00000011166|Gasterosteus_aculeatus|Myod1
ENSONIP00000013575|Oreochromis_niloticus|Myod1
ENSTRUP00000042484|Takifugu_rubripes|Myod1
ENSTNIP00000005370|Tetraodon_nigroviridis|Myod1
ENSONIP00000009955|Oreochromis_niloticus|Myod
ENSTNIP00000021976|Tetraodon_nigroviridis|Myod
ENSGACP00000022921|Gasterosteus_aculeatus|Myod
ENSP00000250003|Homo_sapiens|MYOD1
0,98
0,52
0,91
0,67
0,79
0,97
1
1
0,94
0.6

Pik3ca

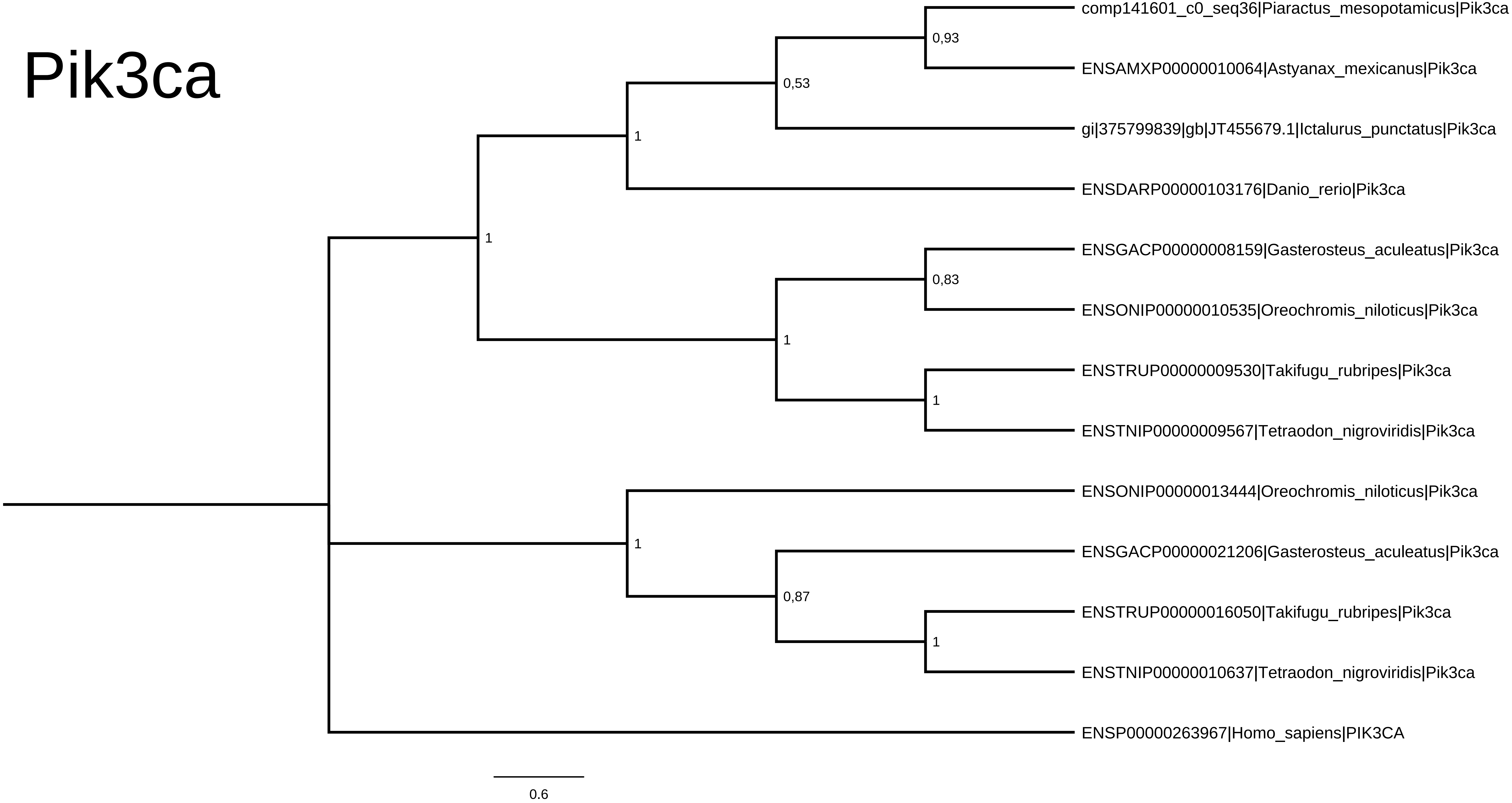

comp141601_c0_seq36|Piaractus_mesopotamicus|Pik3ca
0,93
ENSAMXP00000010064|Astyanax_mexicanus|Pik3ca
0,53
gi|375799839|gb|JT455679.1|Ictalurus_punctatus|Pik3ca
1
ENSDARP00000103176|Danio_rerio|Pik3ca
1
ENSGACP00000008159|Gasterosteus_aculeatus|Pik3ca
0,83
ENSONIP00000010535|Oreochromis_niloticus|Pik3ca
1
ENSTRUP00000009530|Takifugu_rubripes|Pik3ca
1
ENSTNIP00000009567|Tetraodon_nigroviridis|Pik3ca
ENSONIP00000013444|Oreochromis_niloticus|Pik3ca
1
ENSGACP00000021206|Gasterosteus_aculeatus|Pik3ca
0,87
ENSTRUP00000016050|Takifugu_rubripes|Pik3ca
1
ENSTNIP00000010637|Tetraodon_nigroviridis|Pik3ca
ENSP00000263967|Homo_sapiens|PIK3CA
0.6

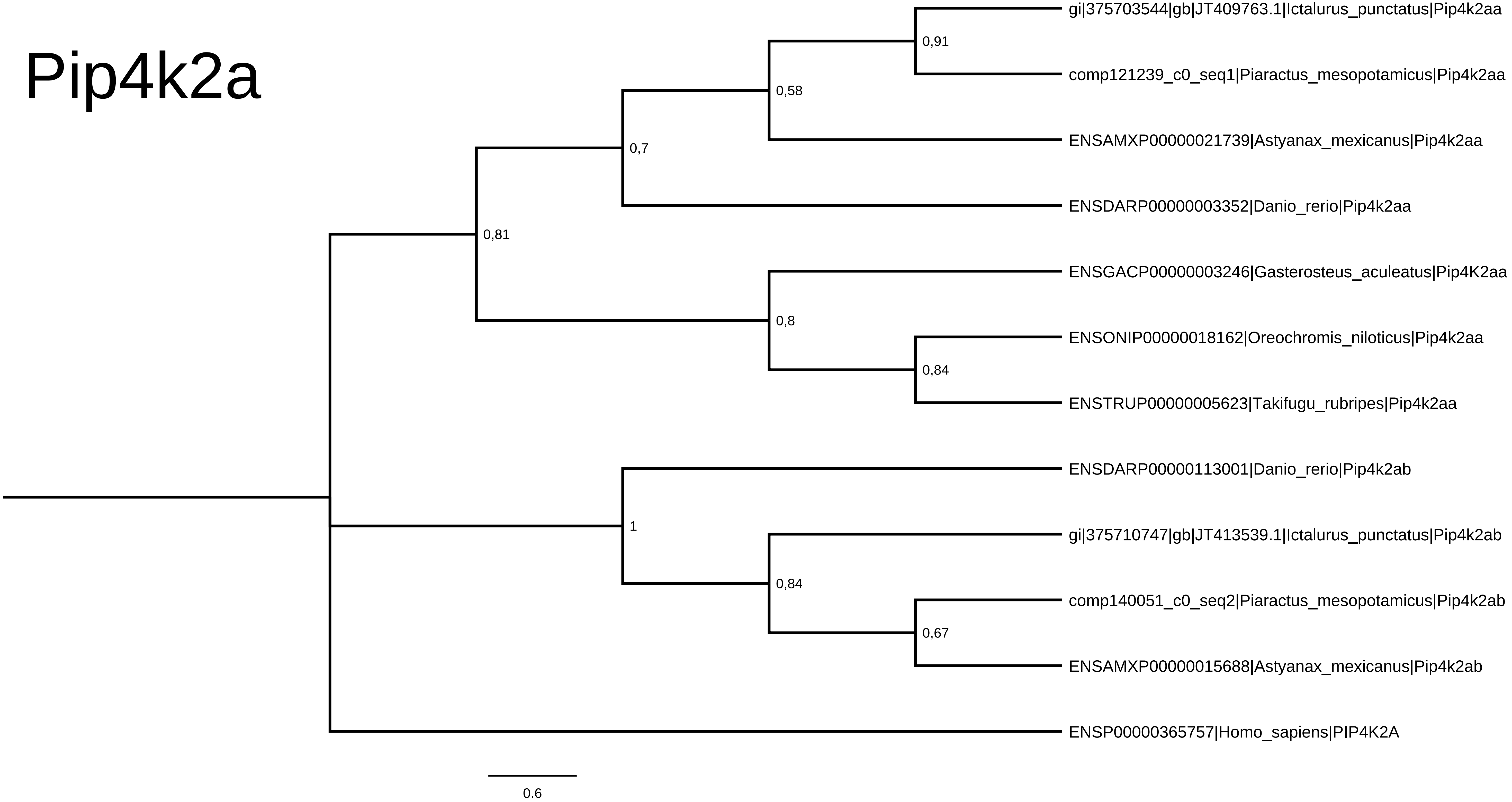
Pip4k2a
gi|375703544|gb|JT409763.1|Ictalurus_punctatus|Pip4k2aa
0,91
comp121239_c0_seq1|Piaractus_mesopotamicus|Pip4k2aa
0,58
ENSAMXP00000021739|Astyanax_mexicanus|Pip4k2aa
0,7
ENSDARP00000003352|Danio_rerio|Pip4k2aa
0,81
ENSGACP00000003246|Gasterosteus_aculeatus|Pip4K2aa
0,8
ENSONIP00000018162|Oreochromis_niloticus|Pip4k2aa
0,84
ENSTRUP00000005623|Takifugu_rubripes|Pip4k2aa
ENSDARP00000113001|Danio_rerio|Pip4k2ab
1
gi|375710747|gb|JT413539.1|Ictalurus_punctatus|Pip4k2ab
0,84
comp140051_c0_seq2|Piaractus_mesopotamicus|Pip4k2ab
0,67
ENSAMXP00000015688|Astyanax_mexicanus|Pip4k2ab
ENSP00000365757|Homo_sapiens|PIP4K2A
0.6

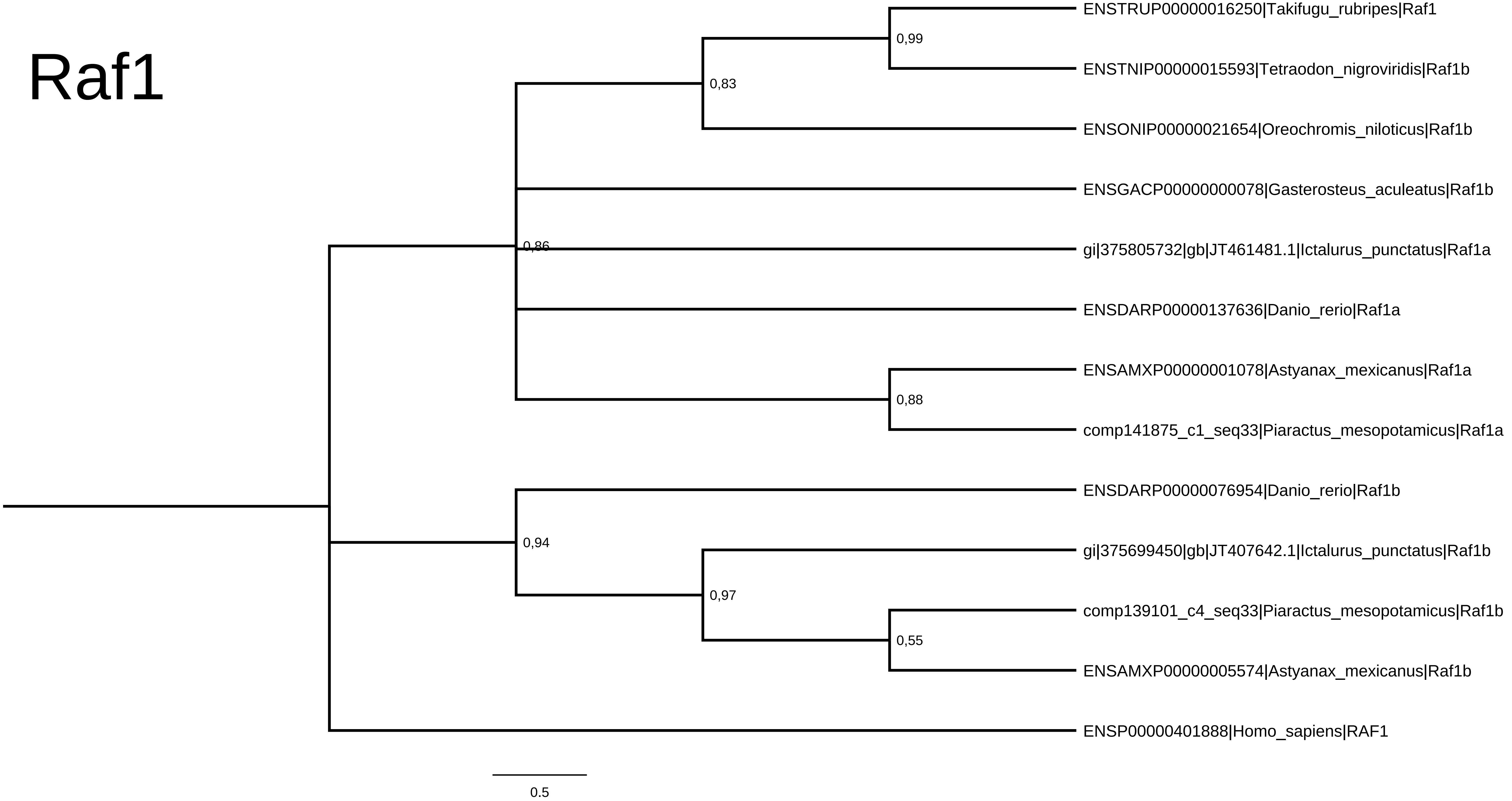

Raf1
ENSTRUP00000016250|Takifugu_rubripes|Raf1
0,99
ENSTNIP00000015593|Tetraodon_nigroviridis|Raf1b
0,83
ENSONIP00000021654|Oreochromis_niloticus|Raf1b
ENSGACP00000000078|Gasterosteus_aculeatus|Raf1b
0,86
gi|375805732|gb|JT461481.1|Ictalurus_punctatus|Raf1a
ENSDARP00000137636|Danio_rerio|Raf1a
ENSAMXP00000001078|Astyanax_mexicanus|Raf1a
0,88
comp141875_c1_seq33|Piaractus_mesopotamicus|Raf1a
ENSDARP00000076954|Danio_rerio|Raf1b
0,94
gi|375699450|gb|JT407642.1|Ictalurus_punctatus|Raf1b
0,97
comp139101_c4_seq33|Piaractus_mesopotamicus|Raf1b
0,55
ENSAMXP00000005574|Astyanax_mexicanus|Raf1b
ENSP00000401888|Homo_sapiens|RAF1
0.5

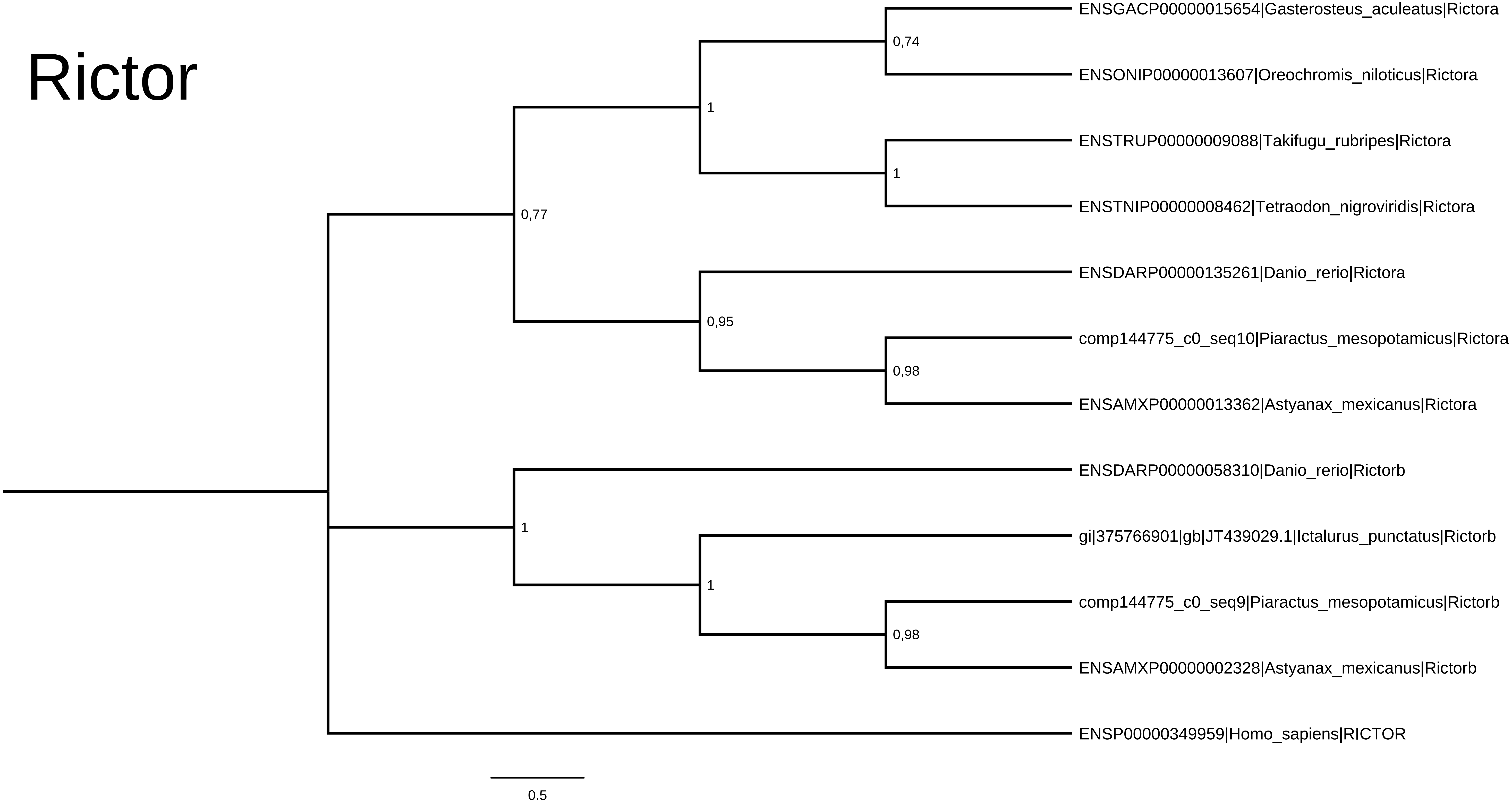
Rictor
ENSGACP00000015654|Gasterosteus_aculeatus|Rictora
0,74
ENSONIP00000013607|Oreochromis_niloticus|Rictora
1
ENSTRUP00000009088|Takifugu_rubripes|Rictora
1
ENSTNIP00000008462|Tetraodon_nigroviridis|Rictora
0,77
ENSDARP00000135261|Danio_rerio|Rictora
0,95
comp144775_c0_seq10|Piaractus_mesopotamicus|Rictora
0,98
ENSAMXP00000013362|Astyanax_mexicanus|Rictora
ENSDARP00000058310|Danio_rerio|Rictorb
1
gi|375766901|gb|JT439029.1|Ictalurus_punctatus|Rictorb
1
comp144775_c0_seq9|Piaractus_mesopotamicus|Rictorb
0,98
ENSAMXP00000002328|Astyanax_mexicanus|Rictorb
ENSP00000349959|Homo_sapiens|RICTOR
0.5

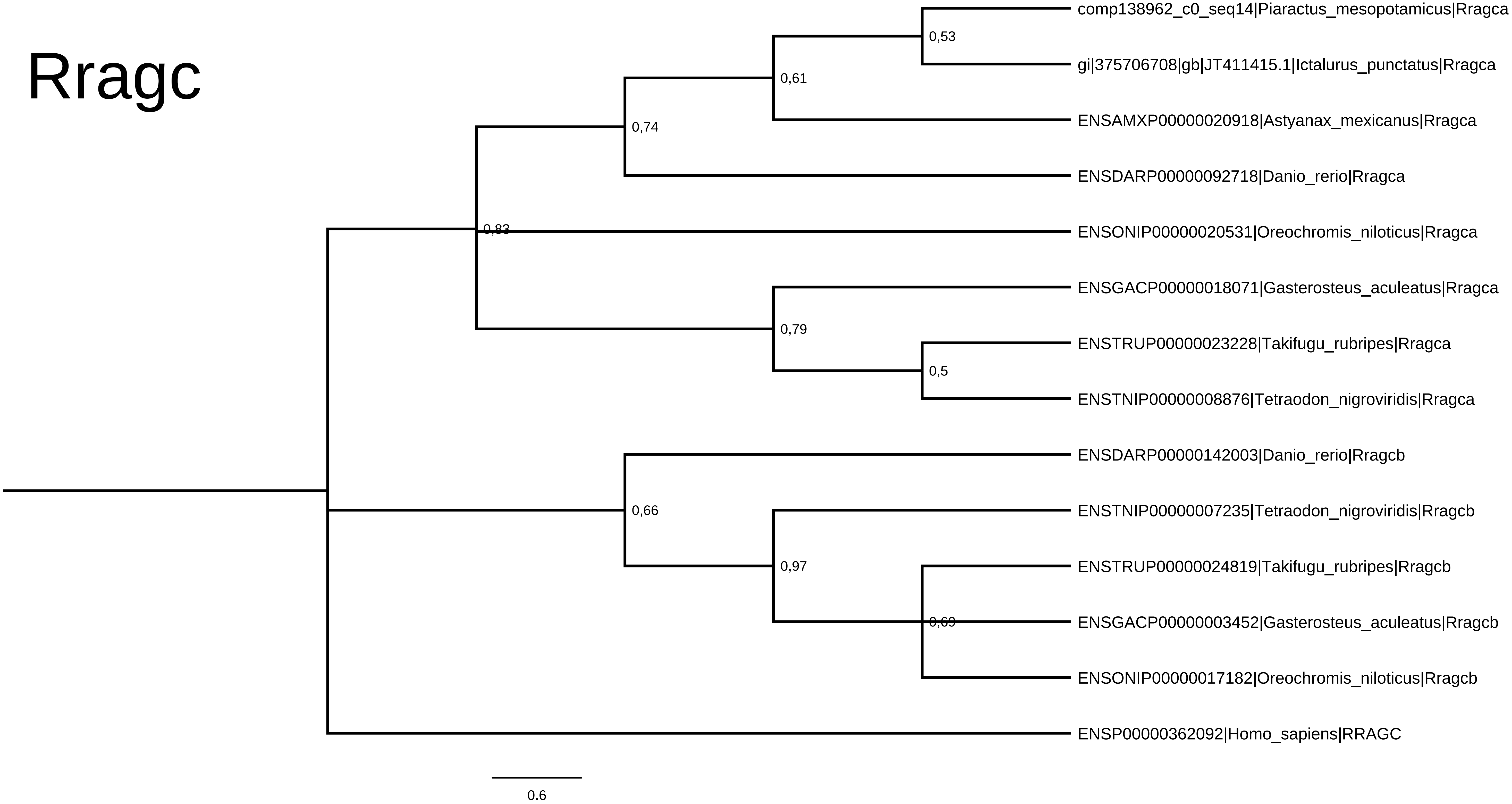

Rragc
comp138962_c0_seq14|Piaractus_mesopotamicus|Rragca
gi|375706708|gb|JT411415.1|Ictalurus_punctatus|Rragca
ENSAMXP00000020918|Astyanax_mexicanus|Rragca
ENSDARP00000092718|Danio_rerio|Rragca
ENSONIP00000020531|Oreochromis_niloticus|Rragca
ENSGACP00000018071|Gasterosteus_aculeatus|Rragca
ENSTRUP00000023228|Takifugu_rubripes|Rragca
ENSTNIP00000008876|Tetraodon_nigroviridis|Rragca
ENSDARP00000142003|Danio_rerio|Rragcb
ENSTNIP00000007235|Tetraodon_nigroviridis|Rragcb
ENSTRUP00000024819|Takifugu_rubripes|Rragcb
ENSGACP00000003452|Gasterosteus_aculeatus|Rragcb
ENSONIP00000017182|Oreochromis_niloticus|Rragcb
ENSP00000362092|Homo_sapiens|RRAGC
0,53
0,61
0,74
0,83
0,79
0,5
0,66
0,97
0,69
0.6

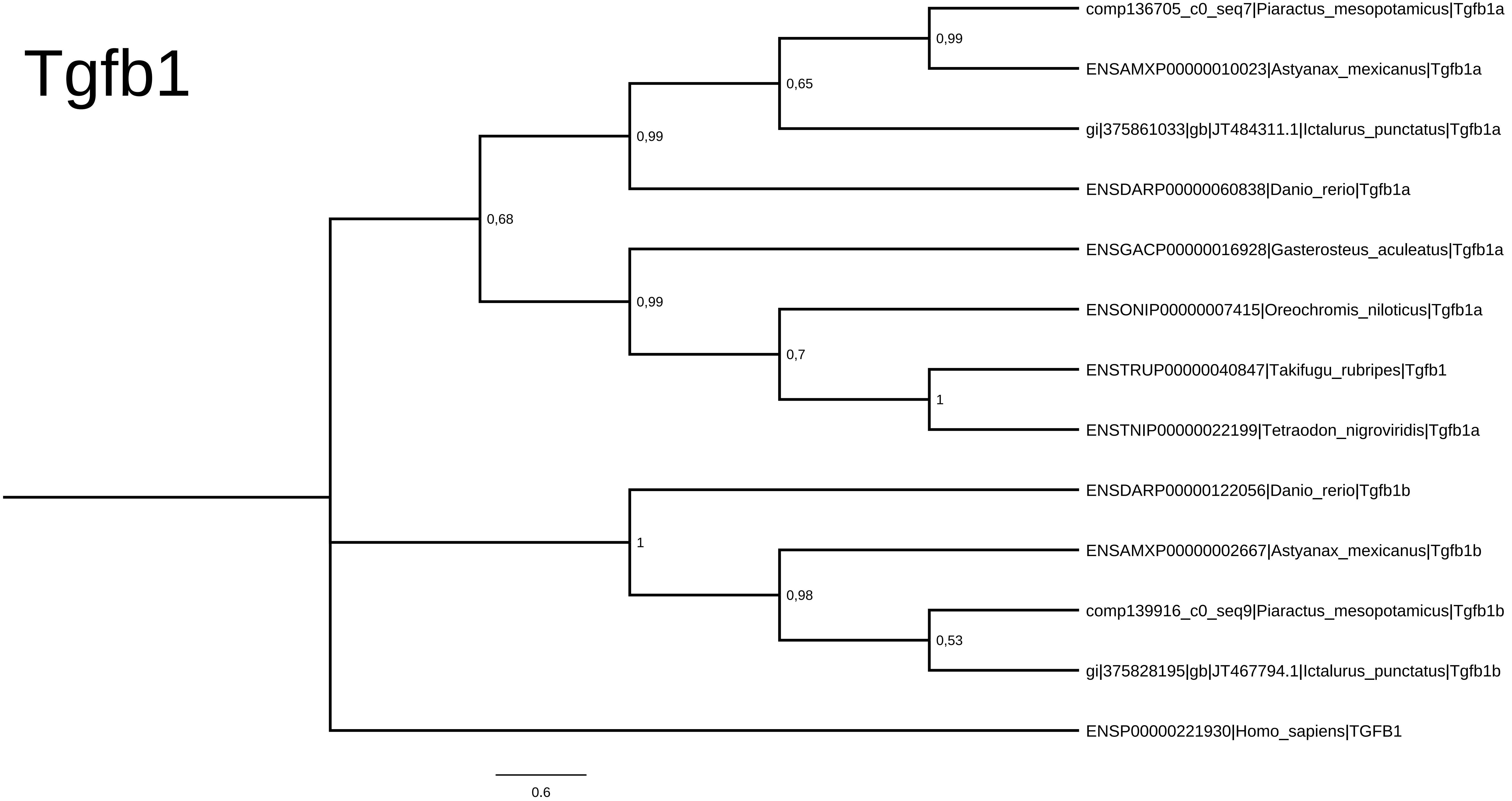
Tgfb1
comp136705_c0_seq7|Piaractus_mesopotamicus|Tgfb1a
0,99
ENSAMXP00000010023|Astyanax_mexicanus|Tgfb1a
0,65
gi|375861033|gb|JT484311.1|Ictalurus_punctatus|Tgfb1a
0,99
ENSDARP00000060838|Danio_rerio|Tgfb1a
0,68
ENSGACP00000016928|Gasterosteus_aculeatus|Tgfb1a
0,99
ENSONIP00000007415|Oreochromis_niloticus|Tgfb1a
0,7
ENSTRUP00000040847|Takifugu_rubripes|Tgfb1
1
ENSTNIP00000022199|Tetraodon_nigroviridis|Tgfb1a
ENSDARP00000122056|Danio_rerio|Tgfb1b
1
ENSAMXP00000002667|Astyanax_mexicanus|Tgfb1b
0,98
comp139916_c0_seq9|Piaractus_mesopotamicus|Tgfb1b
0,53
gi|375828195|gb|JT467794.1|Ictalurus_punctatus|Tgfb1b
ENSP00000221930|Homo_sapiens|TGFB1
0.6

Tgfb3

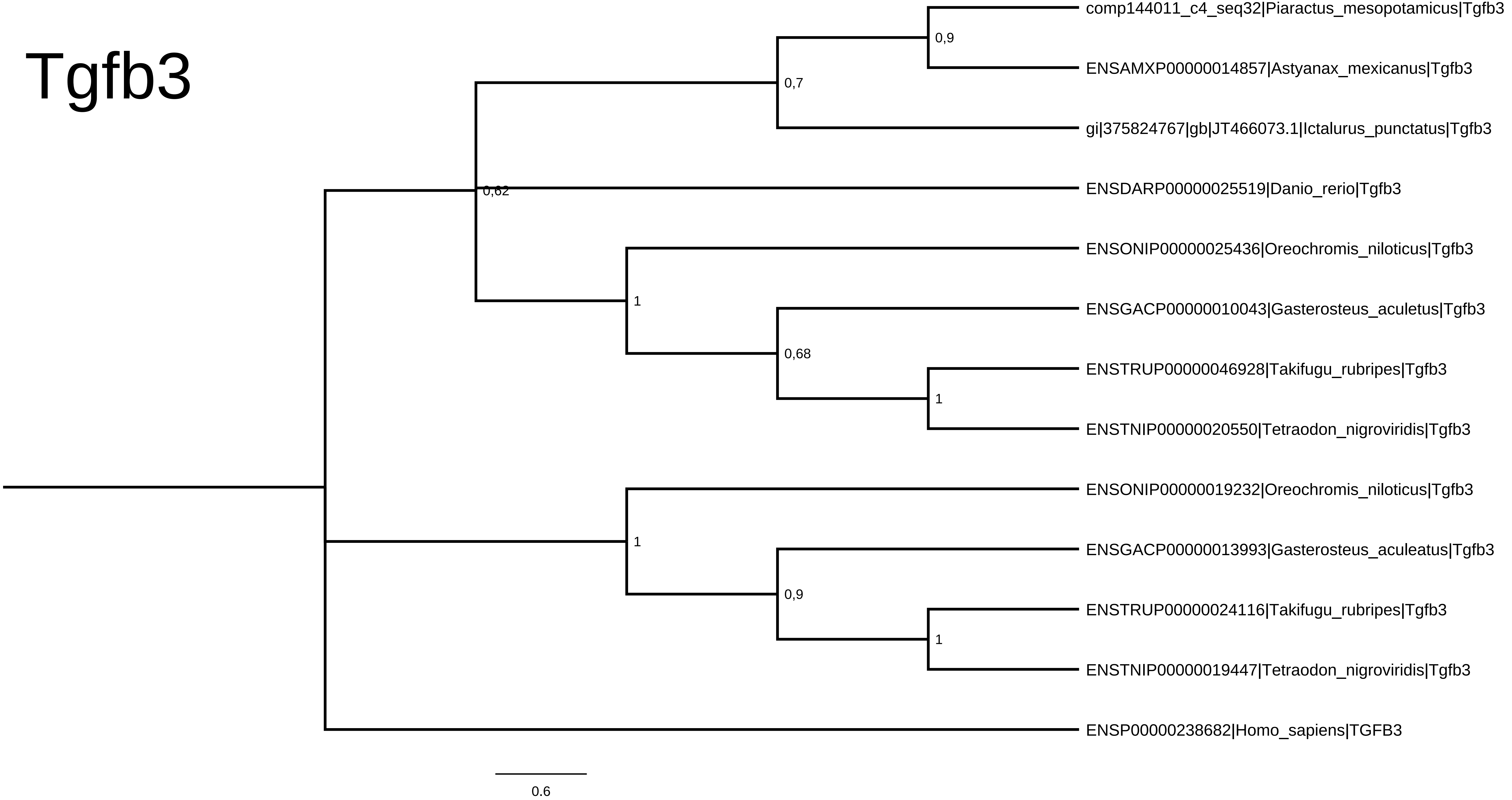
comp144011_c4_seq32|Piaractus_mesopotamicus|Tgfb3
0,9
ENSAMXP00000014857|Astyanax_mexicanus|Tgfb3
0,7
gi|375824767|gb|JT466073.1|Ictalurus_punctatus|Tgfb3
0,62
ENSDARP00000025519|Danio_rerio|Tgfb3
ENSONIP00000025436|Oreochromis_niloticus|Tgfb3
1
ENSGACP00000010043|Gasterosteus_aculetus|Tgfb3
0,68
ENSTRUP00000046928|Takifugu_rubripes|Tgfb3
1
ENSTNIP00000020550|Tetraodon_nigroviridis|Tgfb3
ENSONIP00000019232|Oreochromis_niloticus|Tgfb3
1
ENSGACP00000013993|Gasterosteus_aculeatus|Tgfb3
0,9
ENSTRUP00000024116|Takifugu_rubripes|Tgfb3
1
ENSTNIP00000019447|Tetraodon_nigroviridis|Tgfb3
ENSP00000238682|Homo_sapiens|TGFB3
0.6

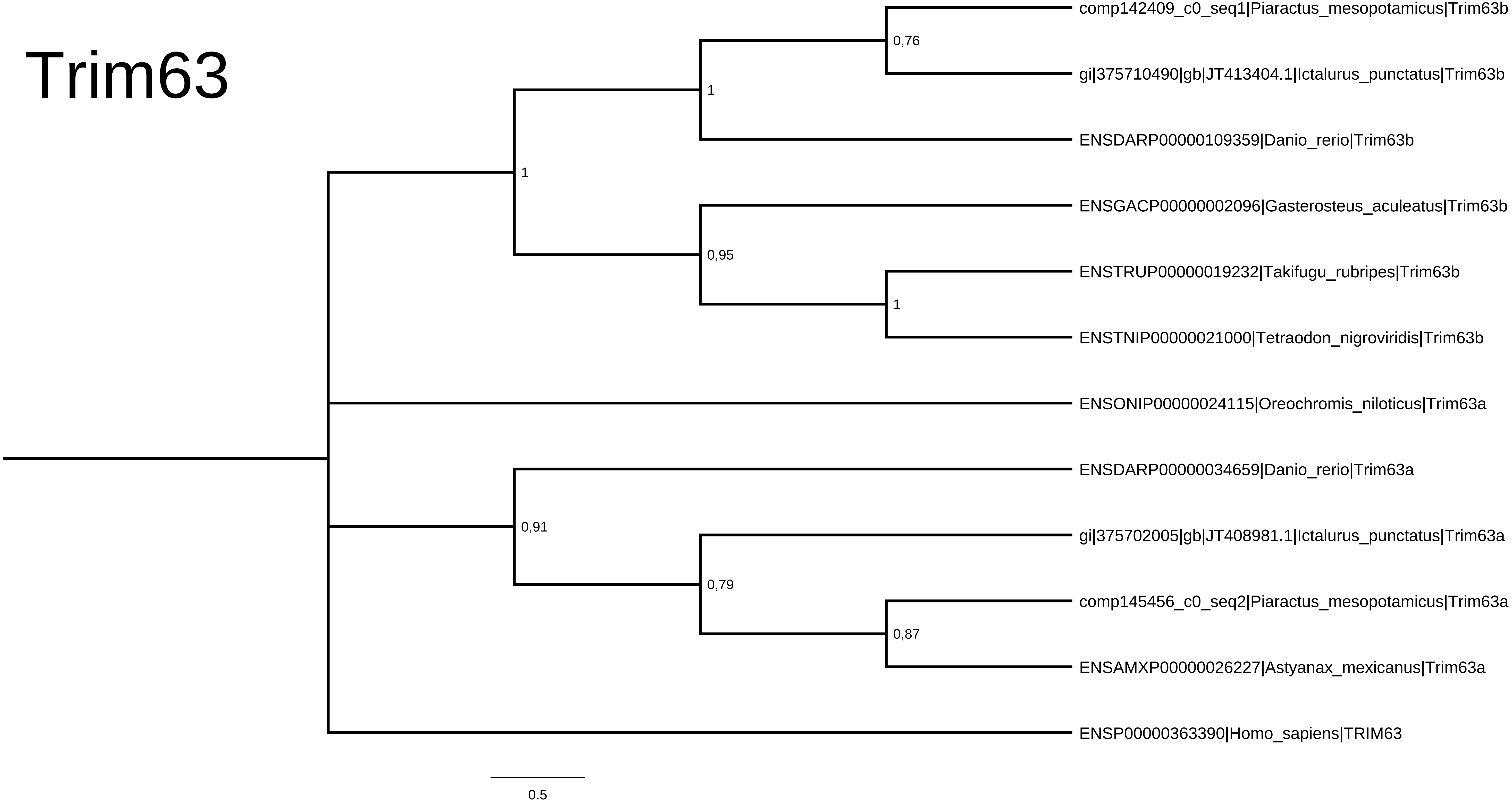
Trim63
comp142409_c0_seq1|Piaractus_mesopotamicus|Trim63b
0,76
gi|375710490|gb|JT413404.1|Ictalurus_punctatus|Trim63b
1
ENSDARP00000109359|Danio_rerio|Trim63b
1
ENSGACP00000002096|Gasterosteus_aculeatus|Trim63b
0,95
ENSTRUP00000019232|Takifugu_rubripes|Trim63b
1
ENSTNIP00000021000|Tetraodon_nigroviridis|Trim63b
ENSONIP00000024115|Oreochromis_niloticus|Trim63a
ENSDARP00000034659|Danio_rerio|Trim63a
0,91
gi|375702005|gb|JT408981.1|Ictalurus_punctatus|Trim63a
0,79
comp145456_c0_seq2|Piaractus_mesopotamicus|Trim63a
0,87
ENSAMXP00000026227|Astyanax_mexicanus|Trim63a
ENSP00000363390|Homo_sapiens|TRIM63
0.5
