# Supplementary material for: An insight on the impact of teleost whole genome duplication on the regulation of the molecular networks controlling skeletal muscle growth
Source: PLoS One. 2021 Jul 22;16(7):e0255006. doi: 10.1371/journal.pone.0255006 (PMC8297816; doi:10.1371/journal.pone.0255006)
Supplement: S3 File — Primers used for qPCR amplification of the LSOs akt2, atf4, cdc42bpa, chuk, eif3j, fst, grb2, igf2, igf2bp2, igfbp3, mef2d, myod, pik3ca, pip4k2a, raf1, rictor, rragc, tgfb1, tgfb3 and trim63. Primers were also designed for the reference genes rpl13, rpl19 and ppiaa, and also for the fbxo32 to prove the efficacy of fasting-refeeding treatments. Accession code based on European Nucleotide Archive—ENA (accession number PRJEB6656) for pacu [60] and Ensembl Genome Browser 102 database (https://www.ensembl.org) for Nile tilapia. (PDF) [file pone.0255006.s003.pdf]

S3 File. LSOs accession codes, Real time PCR primer sequences and PCR efficiencies. Primers used for qPCR amplification of the LSOs *akt2*, *atf4*, *cdc42bpa*, *chuk*, *eif3j*, *fst*, *grb2*, *igf2*, *igf2bp2*, *igfbp3*, *mef2d*, *myod*, *pik3ca*, *pip4k2a*, *raf1*, *rictor*, *rragc*, *tgfb1*, *tgfb3* and *trim63*. Primers were also designed for the reference genes *rpl13*, *rpl19* and *ppiaa*, and also for the *fbxo32* to prove the efficacy of fasting-refeeding treatments. Accession code based on European Nucleotide Archive - ENA (accession number PRJEB6656) for pacu (Mareco et al., 2015) and Ensembl Genome Browser 102 database (https://www.ensembl.org) for Nile tilapia.

Gene	Accession Code	Primers (5' to 3')	E (%)	Tm (°C)
akt2 pacu	comp144275_c3_seq41	F: CCAATGCTGAGAGGGAAGAG<br>R: GTTGATGTCCATTGGCTCCT	96	80
akt2a tilapia	ENSONIP00000006025	F: AACCAATGGATGTTCTCAGC<br>R: GGACATAACCGCTTCCATCT	102	75
akt2b tilapia	ENSONIP00000006516	F: CTCCCCGAGTGACAGCAGTGG<br>R: CTGTTGCCTTCTCCTTCACC	99	81
atf4 pacu	comp145578_c0_seq1	F: GGAGCAAGAACCACCACAAT<br>R: GGGCAGTGAAGTGGACATCT	104	84
atf4a tilapia	ENSONIP00000016467	F: GTTGGAGCAGATGATGGCA<br>R: TCCAATGAAAGACTCCAGGTC	97	77
atf4b tilapia	ENSONIP00000010752	F: GGATGTGCTCAGTGACTTGG<br>R: CAGTGAGTCCAGGTCAAGGTC	99	77
cdc42bpaa pacu	comp123183_c0_seq3	F: GGCACTCTGACTTGTCCCTC<br>R: ACCTCTCTGATGGTGGGACT	96	83
cdc42bpab pacu	comp145167_c3_seq39	F: GATCTTCCAGTCGGGGTC<br>R: CTCCGTCTAACACCCCATCAC	100	84
cdc42bpa tilapia	ENSONIP00000001562	F: GTGAGCGGGAAGAGTTTGAG<br>R: TTTCTTCGGTGAGCAGGACT	98	77
chuk pacu	comp143689_c1_seq38	F: ATCGTTTGTGGGCACTCTTC<br>R: TACGCACTTTGCTTGTCCAC	95	81
chuka tilapia	ENSONIP00000022863	F: GCTGTTGGAGCCGATGGAGG<br>R: AGCAGAGGGGTTTCTTGGTT	96	81
chukb tilapia	ENSONIP00000012095	F: CGGCACAAACAAGCCATACT<br>R: TATCCTCAGCACCCAAAACC	95	78
eif3ja pacu	comp137884_c0_seq67	F: GAGGAATCAAAAGACAGCACAG<br>R: AAGCATCTTTCGCTAGTTCCA	98	77
eif3jb pacu	comp144636_c0_seq88	F: GAGTCCCAGGCTGATGTGC<br>R: GGGACACATGGCTTCAATTC	97	83
eif3j tilapia	ENSONIP00000007868	F: ACAACAGGAGGCAATGGAAC<br>R: AGCTCCAAGTCTGCGTCTTC	97	77
fsta pacu	comp143036_c0_seq11	F: AATGTCCCGAGCCTTCCACT<br>R: CTCTGGCTCTGGCATTTCAC	96	84
fstb pacu	comp144113_c3_seq1	F: CACCACCATTCAGGAGCAG<br>R: GAAGAACGGGAGATGTCAGG	98	84
fst tilapia	ENSONIP00000017943	F: GGATGCTGAAACACCACCTT<br>R: ATGTAGAGCACCTGGCACCT	97	80
grb2a pacu	comp138113_c0_seq16	F: CCCATCTTCCTGAGAGACATC<br>R: GTTAGGGTCCGAGTTGTCCA	105	84

grb2b pacu	comp116716_c1_seq1	F: CCCTGTTTGATTTTGACCCT<br>R: CGTAATTGCGTGGGAACATT	104	83
grb2 tilapia	ENSONIP00000010393	F: TATAGAGCAGGTCCCCCAGA<br>R: ACCAGTTGGGGTCAGAGTTG	97	83
igf2a pacu	comp133875_c0_seq2	F: AGAAGGCTCAAAGGCTCAGG<br>R: CTGTTGGGCACGGTCATC	101	83
igf2b pacu	comp133875_c0_seq1	F: CAACTTCCACAAGCCTCTCATCTC<br>R: CGTAGTCTTCTGTGGGATGC	99	79
igf2 tilapia	ENSONIP00000018238	F: CCCAGCAAAGATACGGACAT<br>R: CCTCTGGACCTTCATTCTGC	100	79
igf2bp2a pacu	comp140394_c4_seq8	F: CCTGATGCTCCTGAGAGAATG<br>R: GCGGGGACTTTGATATGTGT	96	82
igf2bp2b pacu	comp59776_c0_seq2	F: GCTGCGGAGAAACCTATCAC<br>R: AACCTCCTCAGCAGTTTTGG	99	81
igf2bp2 tilapia	ENSONIP00000011355	F: CAGAAGGAAGCCAACGAGAC<br>R: TCCCTGTGTCCTCCTCAATC	103	83
igfbp3 pacu	comp129938_c2_seq1	F: TTCGTAGCCTGGCAGAGACT<br>R: GTTTCCGCATTCCAAACTGT	97	80
igfbp3a tilapia	ENSONIP00000008019	F: CGTTGGAACTGTGAAGGATG<br>R: GCTCTGGGTCTTCCTGTTCTC	105	79
igfbp3b tilapia	ENSONIP00000012146	F: GGGCTCATTTGTCAACATCA<br>R: CATTGACTGGCACTGTTGGT	96	81
mef2d pacu	comp141550_c0_seq12	F: CTTGCTGCTTTGTGAGGTGA<br>R: CCTCCTCTCTGTCTCCAACG	96	84
mef2da tilapia	ENSONIP00000024882	F: GACCAAAGTCCCCTTAATGATG<br>R: TTGACTGTCCGTAGCGTTTG	99	76
mef2db tilapia	ENSONIP00000001942	F: AAAGCCCGCTAAATGACCG<br>R: ATACTGGCACGGTGACTGG	99	83
myod pacu	comp144727_c1_seq4	F: GTTCGTCGTCTTCCTCTTGC<br>R: ACCCGTGCTTTAACACCAAC	101	88
myod1 tilapia	ENSONIP00000013575	F: GACAGCAGCTCTTATTTCTC<br>R: GCTGCTGTTATCGGTGGAGAT	101	83
myod2 tilapia	ENSONIP00000009955	F: GCTCTGATGGCTTGGTGG<br>R: CAATGCTGGACAAACAGTCC	92	80
pik3ca pacu	comp141601_c0_seq36	F: ACGAATGCCAAACCTTATGC<br>R: CGTATCCTCAGGGTGCTGTT	95	83
pik3caa tilapia	ENSONIP00000010535	F: TCTCAGGGCAGAGCAATCG<br>R: TGTCTCAACTGCTCGGTGTC	100	79
pik3cab tilapia	ENSONIP00000013444	F: CTCCCCTGTCAAGTTTCCTG<br>R: CTGTCAGTCAGGGGGTTGTC	95	80
pip4k2aa pacu	comp121239_c0_seq1	F: TTCGTGCTAATGTCTGTCCG<br>R: CGTTAGCCTGTTAGCCTGTCA	98	83
pip4k2ab pacu	comp140051_c0_seq2	F: CACAAGTGAGCACAAAGCG<br>R: GTCTCCCAGCAACAGCCTAA	101	82
pip4k2a tilapia	ENSONIP00000018162	F: TGTCTTCTCTCACCGCCTTT<br>R: ATCTTCTGCCCATCGTTGAT	99	80
raf1a pacu	comp141875_c1_seq33	F: CTAAGCAATGGGCTAGGAATG<br>R: GAATGGTGCTGCTTGTTTTG	98	81
raf1b pacu	comp139101_c4_seq33	F: CCTCAGTAATGGGTTTGGCT<br>R: GAGGAGCGTCGTTGATAAGG	97	78
raf1 tilapia	ENSONIP00000021654	F: CTGCCTTTCTCCGACCATAG<br>R: TGCTGGTTTGGGAGGTAGAC	95	83
rictora pacu	comp144775_c0_seq10	F: GCGGAGTCTCCAGAGTAACG<br>R: CTTCACTGAGCACAGGGTC	96	83

rictorb pacu	comp144775_c0_seq9	F: CGCAACCGCTTTCTTATCTC<br>R: TTAGGGTCGCTGTTGAGCTT	97	83
rictor tilapia	ENSONIP00000013607	F: AAGAGGCTTCAGCAGCAGAG<br>R: AACCTGCGGGGAGTTAGTCT	95	81
rragc pacu	comp138962_c0_seq14	F: GCAACTGAGGGATGAGCTTC<br>R: CACCAGCGAGCTAATGATGA	99	79
rragca tilapia	ENSONIP00000020531	F: CCTTGTTTCTGGAGAGCAC<br>R: GAAGGTGGGGTCAAAGAAGTC	100	80
rragcb tilapia	ENSONIP00000017182	F: GAGGATAGCCCGACATCAGC<br>R: CCTTCTGTATGGAGGATTTGC	97	80
tgfb1a pacu	comp136705_c0_seq7	F: TAGAGGATTGGAAGGGGGTC<br>R: CCAAAGGATACTTCGCCAAC	97	81
tgfb1b pacu	comp139916_c0_seq9	F: CTTGAGGTAAGCTGACTGTGGC<br>R: CAAAGACTTTCGCCTCCAAA	97	80
tgfb1 tilapia	ENSONIP00000007415	F: TGTGGACTTGGAGATGGTGA<br>R: TTCTGCTCCTCATCCTGCTT	100	82
tgfb3 pacu	comp144011_c4_seq32	F: AACAGTCGTCCTTGGTGTCC<br>R: GCAACTTGTCTTGGGCATTT	103	79
tgfb3a tilapia	ENSONIP00000025436	F: CCAGTCCGCCTCAAACAAC<br>R: GTTATCCTGTCCGCAACTCTG	105	81
tgfb3b tilapia	ENSONIP00000019232	F: CCCCTATCAAATCCAAGCG<br>R: TTGTCTTGACCGCAACTCTG	96	79
trim63a pacu	comp145456_c0_seq2	F: ATGTTGCTGTTGTCCATACTCTG<br>R: ATCACATCACCCAGGAGCA	97	82
trim63b pacu	comp142409_c0_seq1	F: GGCTCGGGTCTCTCCTCGG<br>R: CCAGGCGTTACGGAGACC	102	87
trim63 tilapia	ENSONIP00000024115	F: GTTGTCATCCTTCCGTGTCAG<br>R: TCTGAGAGGCGGTAGGTGTTC	96	82
rpl13 pacu	comp141862_c0_seq1	F: ATCAACAGGAAAGTAGCCC<br>R: AGGATGAGTTTGGAGCGGTA	103	82
rpl13 tilapia	ENSONIP00000007470	F: GAATGGAATGATCCTGAACC<br>R: CAGAACGAACCTTGGTATGG	103	85
rpl19 pacu	comp145235_c0_seq2	F: GCAAACTGGTGAAGGATGGT<br>R: CTTGGACTCCCTGTAACGCC	99	87
rpl19 tilapia	ENSONIP00000020477	F: CCCAACGAGACCAATGAGAT<br>R: CGTGCCCTTTCTCTTACCAT	95	83
ppiaa pacu	comp145566_c0_seq1	F: ATTGTGGTTCGTGAAGTCGC<br>R: CCGCTGGGCAGAGTGATTAT	105	85
ppiaa tilapia	ENSONIP00000000799	F: CGGGTCCCAGTTCTTCATC<br>R: GCCGTAGGACTCCATCTTC	105	84
fbxo32 pacu	comp145335_c1_seq5	F: TCTTTGGTGCTCCCCTTGTG<br>R: TAAAACCGAGGACGGCTGG	96	80
fbxo32 tilapia	ENSONIP00000009089	F: CCGAATGGAGAACATCCTG<br>R: GCAGAGTTTCTTCCACAGCA	102	85

F, forward; R, reverse; E, PCR efficiency; Tm, melting temperature.
